# Planar Heptacoordinate Halogens

**DOI:** 10.1021/acsphyschemau.6c00103

**Published:** 2026-08-28

**Authors:** Williams García-Argote, Alejandro Vasquez-Espinal, Lina Ruiz, Luis Leyva-Parra, Gabriel Merino, William Tiznado

**Affiliations:** † Centro de Investigación para el Diseño de Materiales (CEDEM), Facultad de Ciencias Exactas, Departamento de Ciencias Químicas, 28087 Universidad Andres Bello, Avenida República 275, Santiago 8581151, Chile; ‡ Centro de Modelación Ambiental y Dinámica de Sistemas (CEMADIS), Facultad de Ingeniería y Negocios, Universidad de Las Américas, Santiago 7500975, Chile; § Química y Farmacia, Facultad de Ciencias de la Salud, 28035 Universidad Arturo Prat, Casilla 121, Iquique 1100000, Chile; ∥ Instituto de Ciencias Biomédicas, Facultad de Ciencias de la Salud, Universidad Autónoma de Chile, Santiago de Chile 8370146, Chile; ⊥ Departamento de Física Aplicada, 130299 Centro de Investigación y de Estudios Avanzados, Unidad Mérida, Km 6 Antigua Carretera a Progreso, Mérida 97310, Mexico

**Keywords:** planar heptacoordinate halogens, superhalogens, electrostatic confinement, global minima, metal−oxide clusters

## Abstract

Planar hypercoordinate halogens remain challenging because of the high electronegativity of halogens. Through a systematic exploration of 200 X©M_7_E_7_^–^ combinations (X = F–At; M = Be–Ba, Zn–Hg; E = O–Po), we identify four global minima featuring planar heptacoordinate halogens: Br©Zn_7_O_7_^–^, I©Zn_7_O_7_^–^, At©Zn_7_O_7_^–^, and At©Cd_7_O_7_^–^. These systems have large HOMO–LUMO gaps, considerable singlet–triplet separations, and stability up to 1000 K in molecular dynamics simulations. Bonding analyses (WBI, AdNDP, EDA–NOCV, and IQA) indicate that the central halogen behaves as an anion confined within a covalently supported M_7_O_7_ ring, with interactions dominated by electrostatics and minor covalent contributions. Magnetic-response and EDDB analyses do not support aromatic stabilization. Stability instead arises from the combination of a rigid metal–oxide framework and electrostatic confinement. A size-dependent stabilization pattern emerges. Zn-based rings stabilize Br, I, and At, whereas the larger and softer Cd-based framework stabilize only At. Rather than introducing a distinct bonding mechanism, these systems extend the electrostatic confinement mechanism previously established at lower coordination numbers, revealing both its persistence and limitations at *n* = 7. These results show that planar halogen hypercoordination can be sustained up to *n* = 7 only when the cavity size and ring rigidity simultaneously satisfy a narrow electrostatic confinement window, thereby defining the stability limits of the oxide-ring families examined here.

## Graphical abstract

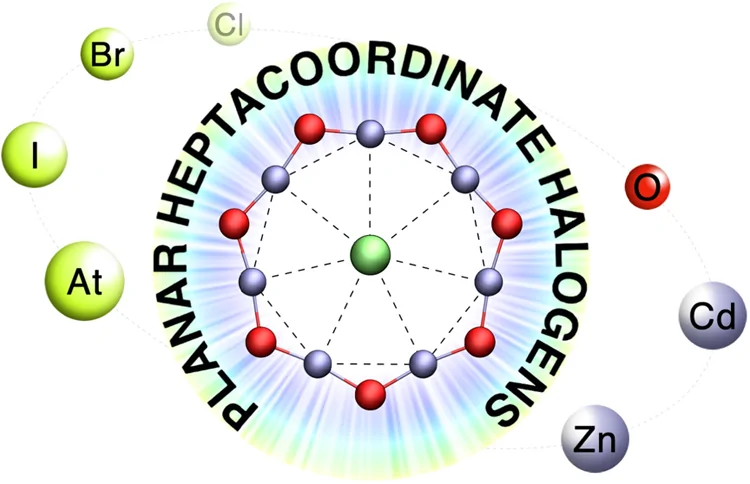

## Introduction

Planar hypercoordinate halogens constitute an emerging area of main-group chemistry, with recent theoretical studies beginning to identify the conditions required to stabilize these motifs.^1–7^ The conceptual basis of planar hypercoordination can be traced to the development of planar tetracoordinate carbon chemistry,^8–11^ which established that planar coordination patterns can be extended to other elements and higher coordination numbers under suitable electronic and geometric conditions.^12–26^ Halogens introduce an additional constraint: their high electronegativity and localized *p* orbitals limit effective in-plane delocalization, favoring terminal or bridging coordination modes over extended planar interactions. Recent work shows that rigid oxide rings can host halogen anions as planar hypercoordinate centers, including F©Zn_5_O_5_^–^ and Cl©Zn_6_O_6_^–^.^27,28^ In these systems, stabilization does not arise from in-plane covalency but from a combination of electrostatic confinement, multicenter interactions, and geometric compatibility between the halogen and the cavity defined by the ring. So, these systems exhibit size-selective behavior. The smaller Zn_5_O_5_ framework accommodates fluorine as a planar pentacoordinate center; expansion to the Zn_6_O_6_ ring destabilizes F©Zn_6_O_6_^–^, resulting in a higher-order saddle point due to the limited interaction of the undersized F atom with the larger cavity. Conversely, larger halogens such as Br are also destabilized in Zn_6_O_6_ due to size mismatch, whereas chlorine provides the balance of size and polarizability required to stabilize a planar hexacoordinate minimum. This size dependence raises a more fundamental question: does the electrostatic confinement mechanism that stabilizes planar hypercoordinate halogens persist as the coordination number increases, or does it break down beyond a certain geometric limit? Addressing this question requires moving beyond isolated examples to evaluate whether the previously identified size-matching principle remains valid as the coordination number increases.

Neutral (MO)_n_ clusters (M = Zn, Cd; *n* ≤ 7) provide a natural platform for this purpose,^29–31^ as they adopt planar ring geometries whose size increases systematically with *n*. In particular, Zn_7_O_7_ represents the largest member of this series that retains a planar structure, providing an opportunity to examine whether expansion of the cavity enables stabilization of heavier halogens in higher coordination environments.

The ability of the host framework to sustain planarity is itself a limiting factor. While Zn_5_O_5_ and Zn_7_O_7_ correspond to global minima and Zn_6_O_6_ lies close in energy to competing structures, previous exploration indicates that Zn_8_O_8_ no longer retains a planar global minimum, suggesting that the structural integrity of the oxide ring imposes an intrinsic upper bound.^30^

Here, we examine whether this stabilization mechanism remains viable at higher coordination numbers by exploring X©M_7_E_7_^–^ systems (X = F–At; M = Be–Ba, Zn–Hg; E = O–Po), designed to map the interplay between cavity size, host rigidity, and halogen identity. Our results identify four global minima featuring a planar heptacoordinate halogen (p7X) center: Br©Zn_7_O_7_^–^, I©Zn_7_O_7_^–^, At©Zn_7_O_7_^–^, and At©Cd_7_O_7_^–^, representing the highest planar coordination number reported to date for a halogen center. These findings indicate a size-dependent selection mechanism: Zn-based rings stabilize Br, I, and At, whereas the larger and softer Cd-based framework stabilizes only At. This study evaluates the extent to which the oxide-ring motif can sustain planar hypercoordination as the cavity expands.

## Methods

The potential energy surfaces (PESs) were explored using the AUTOMATON program.^32^ An initial screening was carried out at the ωB97X-D/SDDAll^33–37^ level, followed by reoptimization of low-energy structures at the ωB97X-D^38^/def2-TZVPP^39^ level with harmonic frequency analysis to confirm their nature as minima or saddle points. All geometry optimizations, frequency calculations, and wave function stability analyses were performed using Gaussian 16 C.02.^40^ Final energies were obtained at the DLPNO–CCSD­(T)^41–43^ /CBS^44,45^ level using the ORCA 6.0 package.^46^

Thermal stability was evaluated through Born–Oppenheimer molecular dynamics (BOMD) simulations at 300, 600, and 1000 K over 50 ps using the PBE0 functional and Stuttgart–Dresden effective core potentials,^47^ as implemented in the deMon2K program.^48,49^

Chemical bonding was characterized using complementary approaches. Wiberg bond indices^50^ (WBI) and natural population analysis^51^ (NPA) were obtained with NBO 7.0,^52^ while Adaptive Natural Density Partitioning^53^ (AdNDP) was performed with Multiwfn 3.8.^54^ Real-space energy decomposition was analyzed through Interacting Quantum Atoms^55–57^ (IQA) at the PBE0-D3 ^59^/def2-TZVPP level using the AIMAll program,^60^ and interaction energies were further examined with energy decomposition analysis using natural orbitals for chemical valence (EDA–NOCV) as implemented in ADF (2024).^61,62^

Magnetic response was evaluated through magnetically induced current densities^63^ (MICD) computed with GIMIC^64^ at the ωB97X-D/def2-TZVPP level using the gauge-including atomic orbital (GIAO) formalism.^65^ Electronic delocalization was quantified using the Electron Density of Delocalized Bonds^66,67^ (EDDB) method at the same level. Full computational details are provided in the ESI.

### Potential Energy Surface Screening

The identification of p7X candidates followed the workflow outlined in Scheme 1. A total of 200 *D*_7*h*_-derived X©M_7_E_7_^–^ combinations (X = F–At; M = Be–Ba, Zn–Hg; E = O–Po) were optimized at the ωB97X-D^38^/def2-TZVPP^39^ level in both singlet and triplet multiplicities. Of these, only eight retain the planar *D*_7*h*_ arrangement as true minima (Tables S1–S2): Br©Zn_7_O_7_^–^, I©Zn_7_O_7_^–^, At©Zn_7_O_7_^–^, and At©Cd_7_O_7_^–^ from the group-12 series, together with Cl©Be_7_O_7_^–^, Br©Mg_7_O_7_^–^, I©Mg_7_O_7_^–^, and At©Mg_7_O_7_^–^ from the alkaline-earth series. The remaining 192 optimized *D*_7*h*_ structures exhibit one or more imaginary frequencies and therefore correspond to saddle points at this level. This sharp selectivity is not solely determined by halogen size but also by the intrinsic stability of the host skeleton. Only oxide-based rings (E = O) produce *D*_7h_ minima, and only with specific metal–halogen size combinations. While the global minima of the bare Zn_5_O_5_ and Zn_7_O_7_ rings adopt *D*_5*h*_ and *D*_7*h*_ symmetries, respectively, and Zn_6_O_6_ is nearly degenerate with alternative structures, the global minimum of Zn_8_O_8_ is not planar.^30^ This loss of planar stability in Zn_8_O_8_ prevents further extension of the electrostatic confinement mechanism beyond *n* = 7.

**Scheme 1:**
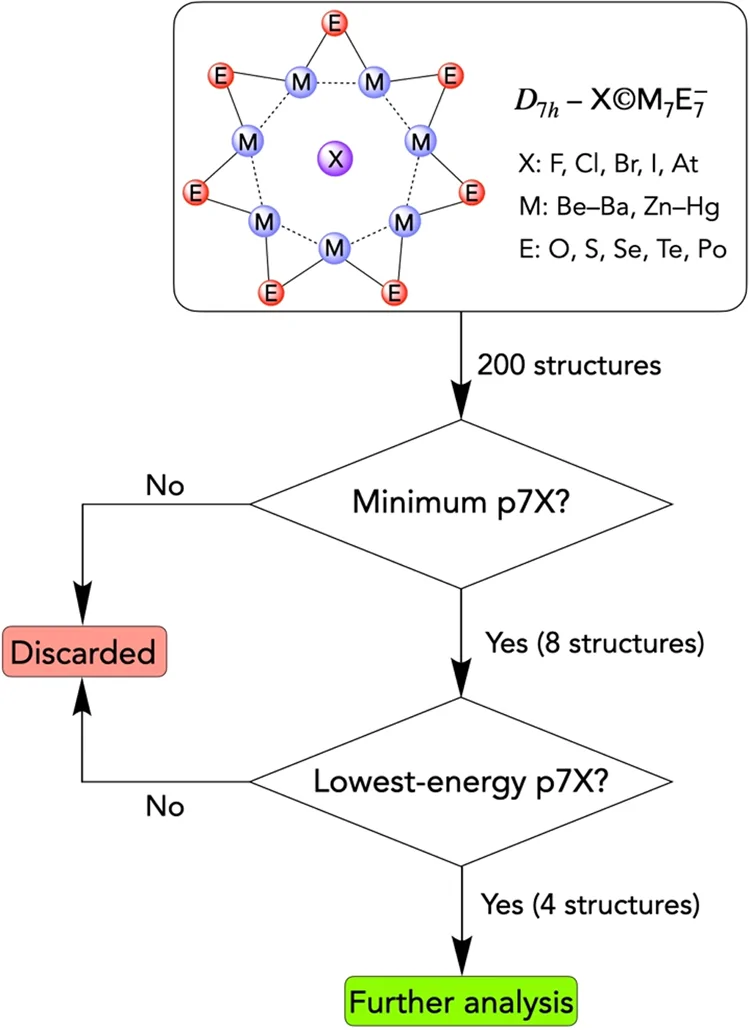
Workflow for Identifying Planar Heptacoordinate Halogen (p7X) Candidates and Their Global Minima from *D*_7*h*_ X©M_7_E_7_^–^ Starting Structures

Potential energy surface (PES) searches on these eight candidates using the AUTOMATON program^32^ identified four of these candidates as global minima: Br©Zn_7_O_7_^–^, I©Zn_7_O_7_^–^, At©Zn_7_O_7_^–^, and At©Cd_7_O_7_^–^. In all cases, the next-lowest isomers correspond to distorted three-dimensional structures, either through displacement of the halogen from the cavity center or partial collapse of the M–O ring. At the DLPNO–CCSD­(T)^41–43^ /CBS^44,45^ //ωB97X-D/def2-TZVPP level, the energy separations from these competing structures are 8.5 (Br©Zn_7_O_7_^–^), 9.9 (I©Zn_7_O_7_^–^), 8.8 (At©Zn_7_O_7_^–^), and 6.1 kcal/mol (At©Cd_7_O_7_^–^), confirming the planar p7X arrangement as the thermodynamically preferred motif (Figures S1–S4). For the At-containing systems, relative energies were also evaluated, including scalar relativistic effects through the zeroth-order regular approximation (ZORA) at the ωB97X-D3/TZ2P-ZORA level on ωB97X-D/def2-TZVPP geometries (Figures S3–S4). In all cases, the energetic ordering among the located isomers is preserved, supporting the global-minimum assignments for At©Zn_7_O_7_^–^ and At©Cd_7_O_7_^–^.

Three of the four global minima are based on the Zn_7_O_7_ framework, whereas At©Cd_7_O_7_^–^ contains Cd instead of Zn. This substitution is associated with the larger cavity of Cd_7_O_7_. The neutral Cd_7_O_7_ ring is itself a global minimum at the ωB97X-D/def2-TZVPP level, lying 1.1 kcal/mol below the next isomer (Figure S5), in line with the known tendency of group-12 metal oxides to form planar ring structures.^29,30^ This result indicates that the anion-in-neutral-ring motif is preserved across the Zn and Cd series, while also suggesting that changes in metal identity provide an additional degree of freedom for stabilizing larger halogens. This sharp reduction (from 200 initial combinations to only four global minima) indicates that planar heptacoordination is not a general outcome of cavity expansion, but rather a highly constrained regime where the electrostatic confinement mechanism operates at its limit.

The electronic structure further supports the intrinsic stability of the identified p7X systems (Table S4). All four species exhibit large HOMO–LUMO gaps (7.5–8.9 eV), substantial singlet–triplet separations (41–58 kcal/mol), and T_1_ diagnostics (0.011–0.015) well below the single-reference threshold (0.02). Harmonic vibrational analyses yield no imaginary frequencies, with the lowest positive frequencies lying in the 4–15 cm^–1^ range, indicating shallow minima at this level. These results indicate that Br©Zn_7_O_7_^–^, I©Zn_7_O_7_^–^, At©Zn_7_O_7_^–^, and At©Cd_7_O_7_^–^ correspond to electronically stable, single-reference ground states containing p7X centers.

The *D*_7*h*_ structures are minima at the ωB97X-D/def2-TZVPP level. Of the tested functionals (Table S3), B3PW91-D3 ^68^ and ωB97X-D, consistently yield positive frequencies for all four systems. In contrast, PBE0-D3, BP86-D3, and TPSSh^58,59,69,70^ produce small imaginary frequencies (|ν| ≤ 14 cm^–1^) for Br©Zn_7_O_7_^–^ and At©Cd_7_O_7_^–^. These low-magnitude imaginary frequencies reveal that the classification of these shallow structures is sensitive to the functional and numerical treatment.

Thermal stability of the planar heptacoordinate structures was evaluated through BOMD simulations at 300, 600, and 1000 K over 50 ps. These simulations probe whether the *D*_7*h*_ arrangement persists under thermal fluctuations or relaxes toward lower-symmetry configurations. The RMSD profile of Br©Zn_7_O_7_^–^ (Figure 1) remains well confined across all temperatures, with mean values of 0.29, 0.37, and 0.47 Å at 300, 600, and 1000 K, respectively. I©Zn_7_O_7_^–^ (Figure S6) exhibits slightly lower RMSD values (0.25, 0.36, and 0.39 Å), corresponding to smaller amplitude structural fluctuations. In both systems, the RMSD increases smoothly with temperature but shows no drift over time, indicating that thermal motion leads to vibrational distortion rather than structural rearrangement.

**Figure 1:**
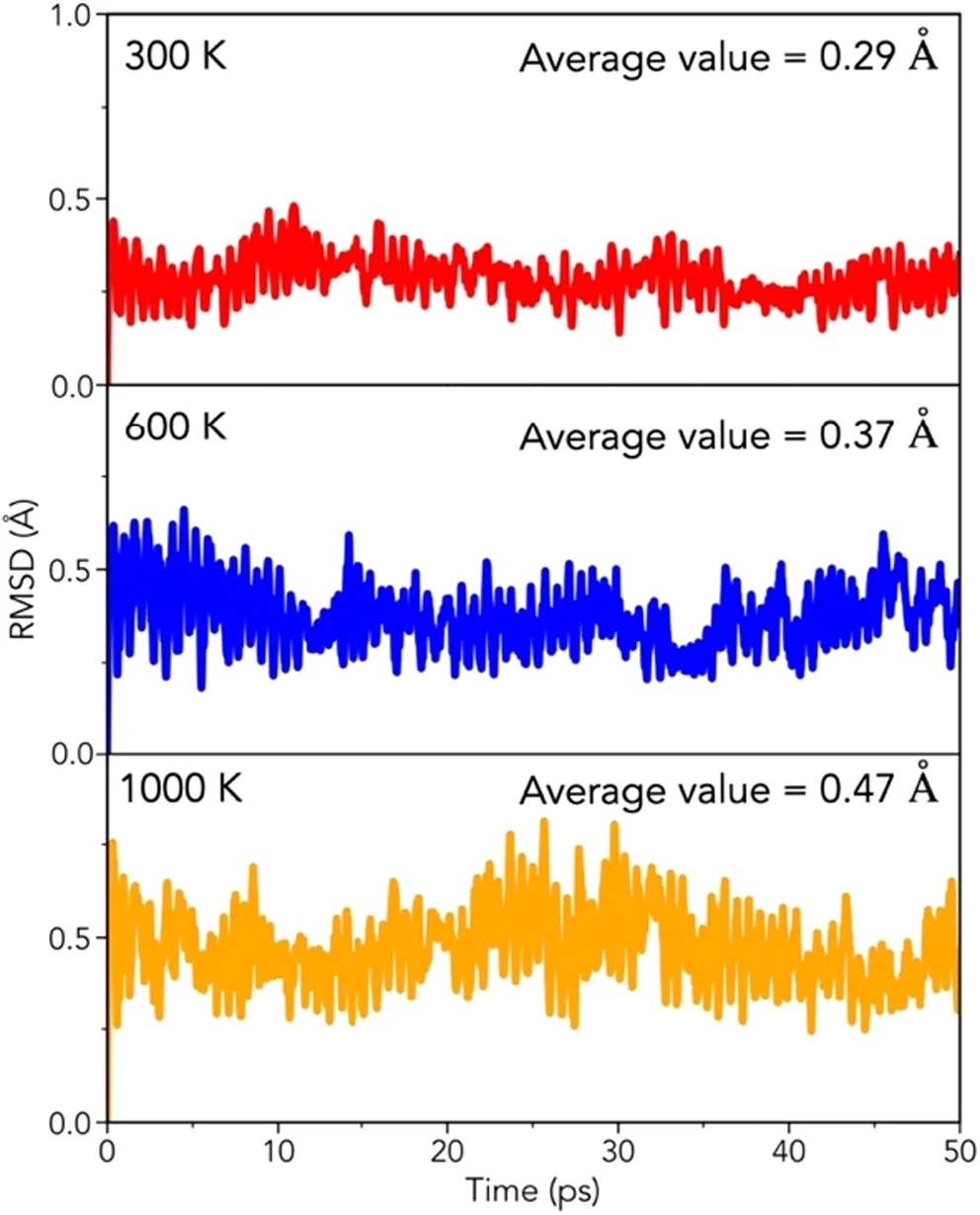
Root-mean-square deviation (RMSD, Å) as a function of simulation time (0–50 ps) for Br©Zn_7_O_7_^–^ from Born–Oppenheimer molecular dynamics (BOMD) simulations at the PBE0/SDD level at 300, 600, and 1000 K.

The At-containing cluster (Figure S7) displays larger RMSD amplitudes, particularly at 1000 K. The larger RMSD amplitudes are consistent with softer vibrational modes and, in the case of At©Cd_7_O_7_^–^, with the greater flexibility of the Cd–O framework. Despite these larger fluctuations, the structural deviations remain bounded, and no transition to alternative geometries is detected. Across all trajectories, no bond cleavage, isomerization, or ring collapse occurs. The halogen remains centered within the cavity, and the metal–oxygen ring retains its integrity. These results indicate that the heptacoordination and the integrity of the metal–oxide rings are retained throughout the 50 ps simulations, even at 1000 K, although appreciable instantaneous deviations from planarity occur.

### Structure and Bonding

The four global minima (Figure 2) share a common structural feature: long X–M distances (3.50–3.52 Å for Zn; 3.89 Å for Cd) that exceed the sums of Pyykkö covalent radii,^71^ together with very small WBI_X–M_ values (0.01–0.02). These descriptors indicate that covalent interactions between the central halogen and the surrounding metal atoms are weak.

**Figure 2:**
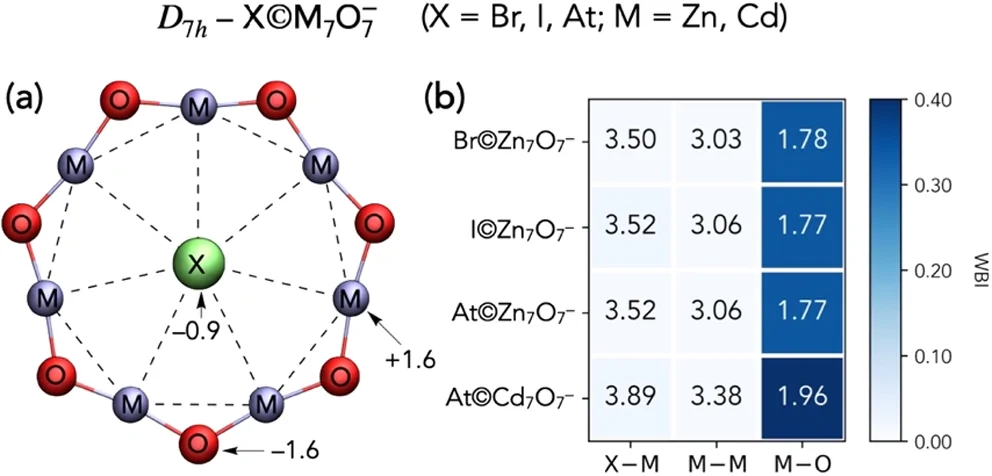
(a) Structure of *D*_7*h*_ planar heptacoordinate halogen systems, X©M_7_O_7_^–^ (X = Br, I, At; M = Zn, Cd), with selected NPA atomic charges |e|. (b) Structural and bonding descriptors: X–M, M–M, and M–O distances (Å) and Wiberg bond indices (WBI).

In contrast, the M_7_O_7_ ring displays short M–O distances (1.77–1.78 Å for Zn; 1.96 Å for Cd) and relatively large WBI_M–O_ values (0.34–0.40), indicating a covalently connected ring. Metal–metal separations are considerably longer (3.03–3.06 Å for Zn; 3.38 Å for Cd), with WBI_M–M_ values close to zero (0.006–0.007), excluding M–M bonding. So, these features define a preorganized, covalently connected ring that remains structurally intact upon incorporation of the central halogen. The charge distribution reinforces this separation of roles. NPA analysis^51^ yields a halide-like center (*q*_X_ ≈ −0.9 |e|), electropositive metal sites (q_M_ ≈ +1.6 |e|), and electronegative oxygen atoms (*q*_O_ ≈ −1.6 |e|). This arrangement (a negative center surrounded by a positively charged inner shell and a negative outer periphery) corresponds to electrostatic confinement of the halogen within the cavity defined by the M_7_O_7_ ring, consistent with an anion-in-neutral-ring motif.

To examine the bonding pattern further, AdNDP analysis^53^ was performed for Br©Zn_7_O_7_^–^ as a representative case (Figure 3). The Zn_7_O_7_ ring is described by seven O 2s lone pairs (ON ≈ 1.99 |e|), 14 2c–2e Zn–O σ bonds (ON ≈ 1.99 |e|), and seven 3c–2e Zn–O–Zn π bonds (ON ≈ 1.99 |e|), matching the covalent ring inferred from WBI values. The central halogen retains four 1c–2e lone pairs (ON ≈ 1.97–1.99 |e|), with no delocalized bonding involving the metal centers, while each Zn atom preserves five 3*d* lone pairs (ON ≈ 1.99 |e|). The same bonding pattern is found for I©Zn_7_O_7_^–^, At©Zn_7_O_7_^–^, and At©Cd_7_O_7_^–^ (Figures S8 and S9), indicating that the halogen remains electronically localized and does not participate in multicenter bonding with the ring.

**Figure 3:**
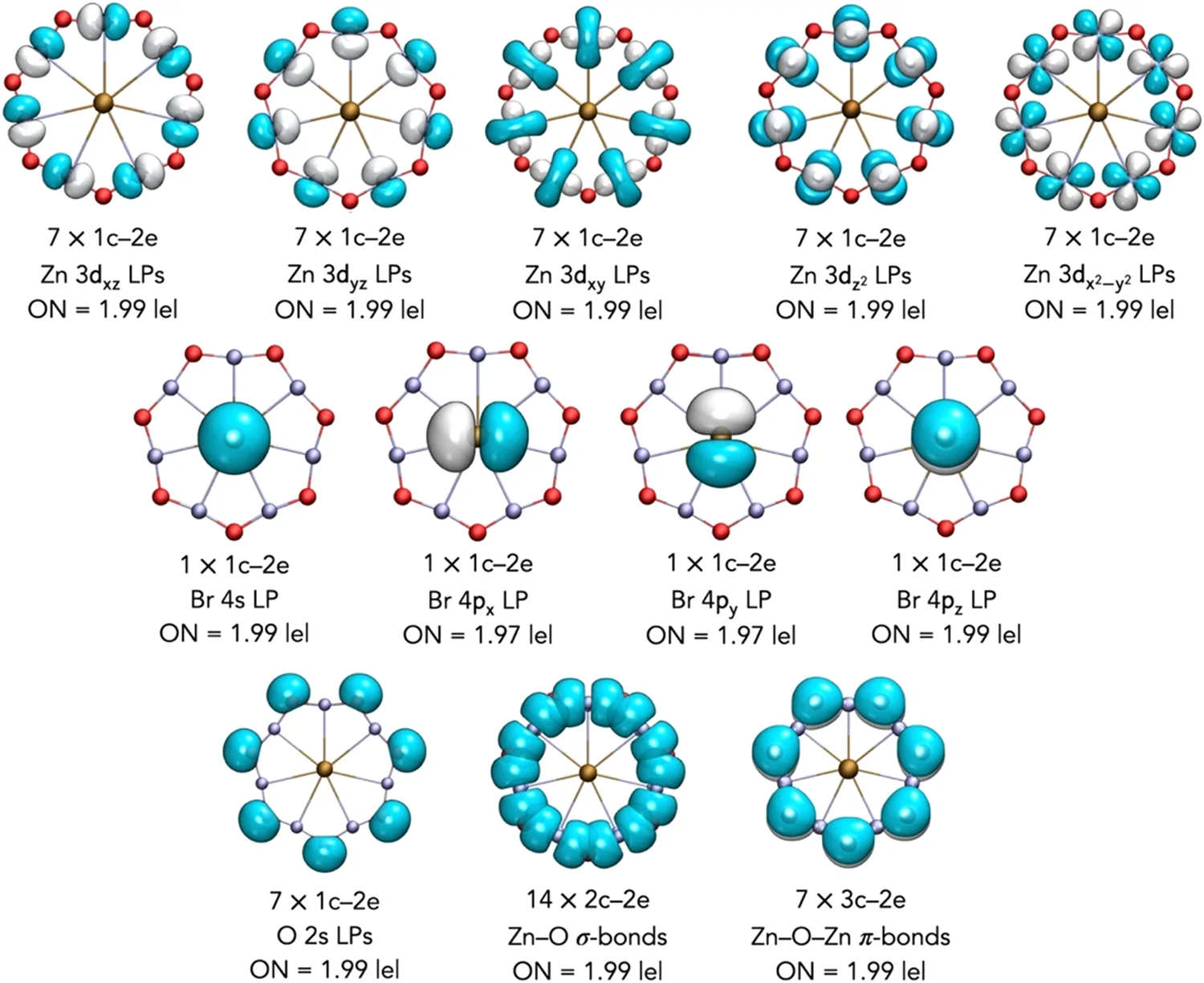
Adaptive Natural Density Partitioning (AdNDP) analysis of Br©Zn_7_O_7_^–^ at the ωB97X-D/def2-TZVPP level. Occupation numbers (ON) are given in |e|.

This description is further supported by the energy decomposition analysis^61^ (EDA–NOCV) performed for all four systems (Tables 1 and S5). Among the tested fragmentation schemes, the X^–^(singlet) + M_7_O_7_(singlet) partition yields the smallest orbital interaction term, providing the most consistent representation of the interaction. The attractive interaction is dominated by the electrostatic component, with Δ*V*_elstat_ accounting for 76–79% and Δ*E*_oi_ contributing 21–24%.

**Table 1:** EDA-NOCV Results for X©Zn_7_O_7_^–^ (X = Br, I, At) using X^–^ and Zn_7_O_7_ as Interacting Fragments at the PBE0/TZ2P-ZORA Level^a^.

**energy terms**	**interaction**	**interaction energies Br** ^**–**^ **(singlet) + Zn**_**7**_ **O**_**7**_ **(singlet)**	**interaction energies I** ^**–**^ **(singlet) + Zn**_**7**_ **O**_**7**_ **(singlet)**	**interaction energies At** ^**–**^ **(singlet) + Zn**_**7**_ **O**_**7**_ **(singlet)**
Δ*E*_int_		–67.2	–61.9	–61.7
Δ*E*_Pauli_		26.1	48.6	61.5
Δ*E*_attr_		–93.3	–110.5	–123.2
Δ*V*_elstat_ ^b^		–70.7 (75.8%)	–86.4 (78.1%)	–96.8 (78.6%)
Δ*E*_oi_ ^b^		–22.6 (24.2%)	–24.1 (21.9%)	–26.4 (21.4%)
Δ*E*_oi(1)_ ^c^	Br^–^/I^–^/At^–^ (4/5/6*p*_*x*_) → Zn_7_O_7_ donation	–6.7 (29.6%)	–7.7 (31.8%)	–8.6 (32.6%)
Δ*E*_oi(2)_ ^c^	Br^–^/I^–^/At^–^ (4/5/6*p*_*y*_) → Zn_7_O_7_ donation	–6.7 (29.6%)	–7.7 (31.8%)	–8.6 (32.6%)
Δ*E*_oi(3)_ ^c^	Br^–^/I^–^/At^–^ (4/5/6*s*) → Zn_7_O_7_ donation	–2.0 (8.8%)	–1.6 (6.7%)	–1.1 (4.2%)
Δ*E*_oi(rest)_ ^c^		–7.2 (32.0%)	–7.2 (29.7%)	–8.1 (30.7%)

a All energy values are given in kcal/mol.

b The percentage contribution with respect to the total attraction is given in parentheses.

c The percentage contribution with respect to the total orbital interaction is given in parentheses.

Within the Zn_7_O_7_ series, the electrostatic contribution increases from Br^–^ (75.8%) to I^–^ (78.1%) to At^–^ (78.6%), indicating a progressive shift toward a more electrostatic interaction as the halogen becomes larger and more polarizable. Decomposition of Δ*E*_oi_ (Figures S10–S12) identifies two dominant degenerate X^–^(*p*_*x*_, *p*_*y*_) → M_7_O_7_ donor channels [Δ*E*_oi(1)_ = Δ*E*_oi(2)_ = −6.7 to −8.6 kcal/mol, 59–65%], together with a weaker X^–^(singlet) → M_7_O_7_ contribution [Δ*E*_oi(3)_ = −1.1 to −2.0 kcal/mol, 4–9%], while the remaining interaction is distributed among higher-order terms [Δ*E*_oi(rest)_ = −7.2 to −8.1 kcal/mol, 30–32%]. These results indicate that the central halogen is best described as an anion confined within a covalently supported M_7_O_7_ ring, with the interaction dominated by electrostatics and only a modest covalent contribution arising from localized lone-pair donation.

To resolve the interaction hierarchy at the atom-pair level, Interacting Quantum Atoms^55–57^ (IQA) analysis was performed (Figure 4). This approach partitions the interaction energy into Coulombic (classical electrostatic) and exchange–correlation (covalent) components, allowing a direct comparison of the nature of X–M, M–O, and M–M interactions within the same framework.

**Figure 4:**
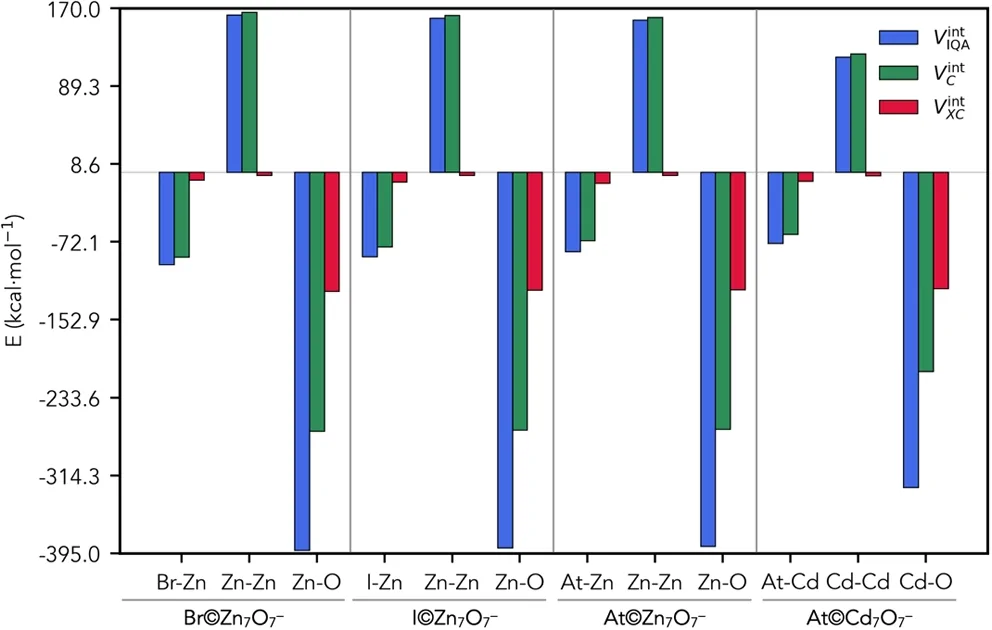
Interacting Quantum Atoms (IQA) energy decomposition for *D*_7*h*_ X©M_7_O_7_^–^ systems (X = Br, I, At; M = Zn, Cd). *V*_IQA_^int^ is the total interatomic interaction energy, decomposed into Coulombic (*V*_C_^int^) and exchange–correlation (*V*_XC_^int^) components (kcal/mol).

The X–M interactions are stabilizing and predominantly Coulombic, with *V*_C_^int^ accounting for 87–92% of the total interaction and *V*_XC_^int^ contributing the remaining 8–13%. This partitioning is consistent with the predominantly electrostatic character indicated by the EDA–NOCV analysis. In contrast, the M–O interactions show a significantly larger exchange–correlation contribution (*V*_C_^int^ = 64–69%; *V*_XC_^int^ = 31–36%), indicating a higher covalent character within the M_7_O_7_ ring. Metal–metal interactions remain repulsive and exhibit negligible exchange–correlation contributions, reinforcing the absence of direct M–M bonding. The IQA delocalization indices provide a complementary real-space measure of electron sharing. The values follow the same hierarchy: δ­(X–M) = 0.08–0.12, δ­(M–O) = 0.84–0.89, and δ­(M–M) and δ­(X–O) ≈ 0.04–0.07. Slightly lower values are obtained for At©Cd_7_O_7_^–^, consistent with its longer interatomic distances.

Because each X–M pair corresponds to a direct stabilizing interaction, the central halogen satisfies the IUPAC definition of coordination number and is therefore heptacoordinate. This interaction pattern does not introduce a new bonding mechanism but rather represents the continuation of the electrostatic confinement regime previously identified in lower-coordinate systems. Its persistence at *n* = 7 indicates that the coordination limit is not determined by the emergence of new bonding modes, but by geometric and electrostatic constraints imposed by the host.

Although the interaction between the halide and the surrounding ring is predominantly electrostatic, the stability of planar hypercoordinate halogens is not determined by electrostatics alone. The geometric match between the size of the halide and the cavity generated by the metal–oxide ring also plays a critical role. The results indicate that the size selectivity in the Zn_n_O_n_ series is controlled by the match between the halide size and the cavity generated by the metal–oxide ring. The Zn–O distances in the isolated rings remain almost unchanged from Zn_5_O_5_ to Zn_7_O_7_ (1.76–1.77 Å), so the cavity grows mainly because the ring contains more Zn–O units. The center-to-Zn distance therefore increases from 2.54 Å in Zn_5_O_5_ to 3.08 Å in Zn_6_O_6_ and 3.63 Å in Zn_7_O_7_. When these cavity sizes are compared with the six-coordinate Shannon radii of the halides (F^–^ = 1.33 Å, Cl^–^ = 1.81 Å, Br^–^ = 1.96 Å, and I^–^ = 2.20 Å), all planar global minima fall within a narrow cavity-to-anion radius ratio of ca. 1.65–1.91, including F©Zn_5_O_5_^–^ (1.91), Cl©Zn_6_O_6_^–^ (1.70), and Br©Zn_7_O_7_^–^ (1.85), and I©Zn_7_O_7_^–^ (1.65), whereas systems outside this range become unstable or lose planarity. For example, if the cavity is too large for the halide, as in F©Zn_6_O_6_^–^ (2.32) or Cl©Zn_7_O_7_^–^ (2.01), the electrostatic confinement becomes too weak and in-plane imaginary modes appear. If the halide is too large for the cavity, as in Br©Zn_6_O_6_^–^ (1.57), the anion is displaced out of the plane. Thus, planar halogen hypercoordination can be sustained to *n* = 7 only when cavity size and ring rigidity jointly satisfy the requirements of a narrow confinement window.

This geometric window is necessary, but it is not sufficient. The framework must also be rigid enough to keep the halide confined. The Zn-based rings adapt moderately upon halide insertion: Zn–O bonds elongate by only 1.0–1.3%, whereas Zn–Zn distances contract by 3.0–5.4%, consistent with a ring that can adjust while maintaining confinement. Although the cavity-to-anion radius ratio of I©Cd_7_O_7_^–^ falls within the range found for several planar global minima, this structure remains a shallow saddle point. Compared with the Zn–O interactions, the Cd–O interactions are less stabilizing according to the IQA analysis, despite retaining appreciable covalent character. Moreover, insertion of I^–^ produces only small structural changes in the Cd_7_O_7_ ring (+0.5% in Cd–O and −2.3% in Cd–Cd), indicating a weaker structural response of the host framework. Thus, the apparently favorable size match is not accompanied by sufficiently strong host–guest coupling to retain I^–^ within the molecular plane. In contrast, the larger At^–^ anion, with an estimated radius of ca. 2.3 Å, interacts more effectively with the wider Cd_7_O_7_ cavity and forms the global-minimum structure. These results show that geometric matching alone is insufficient; planar confinement also requires sufficiently strong interactions between the confined anion and the host framework.

### Electronic Structure

The electronic structure supports the stability of the planar heptacoordinate motif. All four clusters exhibit large HOMO–LUMO gaps (7.5–8.9 eV; Figure 5). The HOMO is predominantly localized on the central halogen (64–70%), whereas the LUMO is centered on the metal oxide framework (66–71%). This spatial separation indicates distinct regions for electron removal and electron addition: detachment primarily involves the confined halide, whereas electron attachment is associated with the M_7_O_7_ ring.

**Figure 5:**
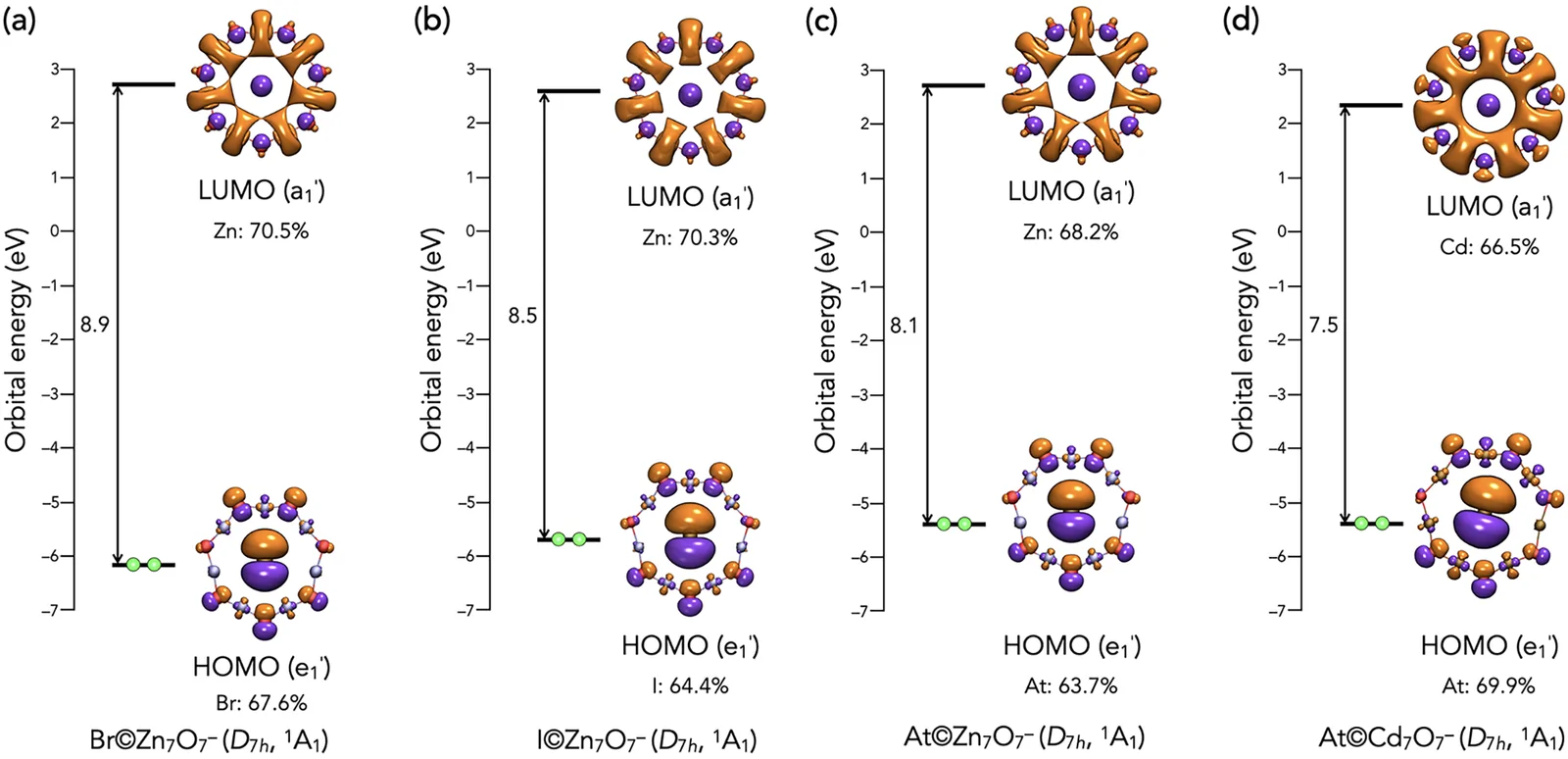
HOMO, LUMO, and HOMO–LUMO gaps for *D*_7*h*_ X©M_7_O_7_^–^ (X = Br, I, At; M = Zn, Cd).

This distribution of frontier orbitals is reflected in the electron-binding strength. The first vertical detachment energies (VDEs; Table S7), computed at the OVGF^72–74^ (5.24–6.39 eV; pole strengths 0.90–0.91) and TD-ωB97X-D (5.50–6.32 eV) levels, exceed the electron affinities of all halogen atoms. These values place the clusters within the superhalogen regime. Within the Zn-based series, both methods follow the trend Br > I > At. Replacing Zn by Cd produces a modest decrease in VDE, as the softer Cd–O framework reduces the electrostatic confinement of the excess electron.

The metal–oxide ring plays a dual role. It defines the cavity that enforces planar heptacoordination and, at the same time, stabilizes the excess electron. Electron binding therefore depends directly on the confinement imposed by the framework.

### Electron Delocalization

Electron delocalization was examined from complementary magnetic-response and electron-density perspectives. Magnetically induced current densities^63^ (MICD) and the EDDB^66,67^ descriptors were computed for all four global minima (Figures 6 and S13).

**Figure 6:**
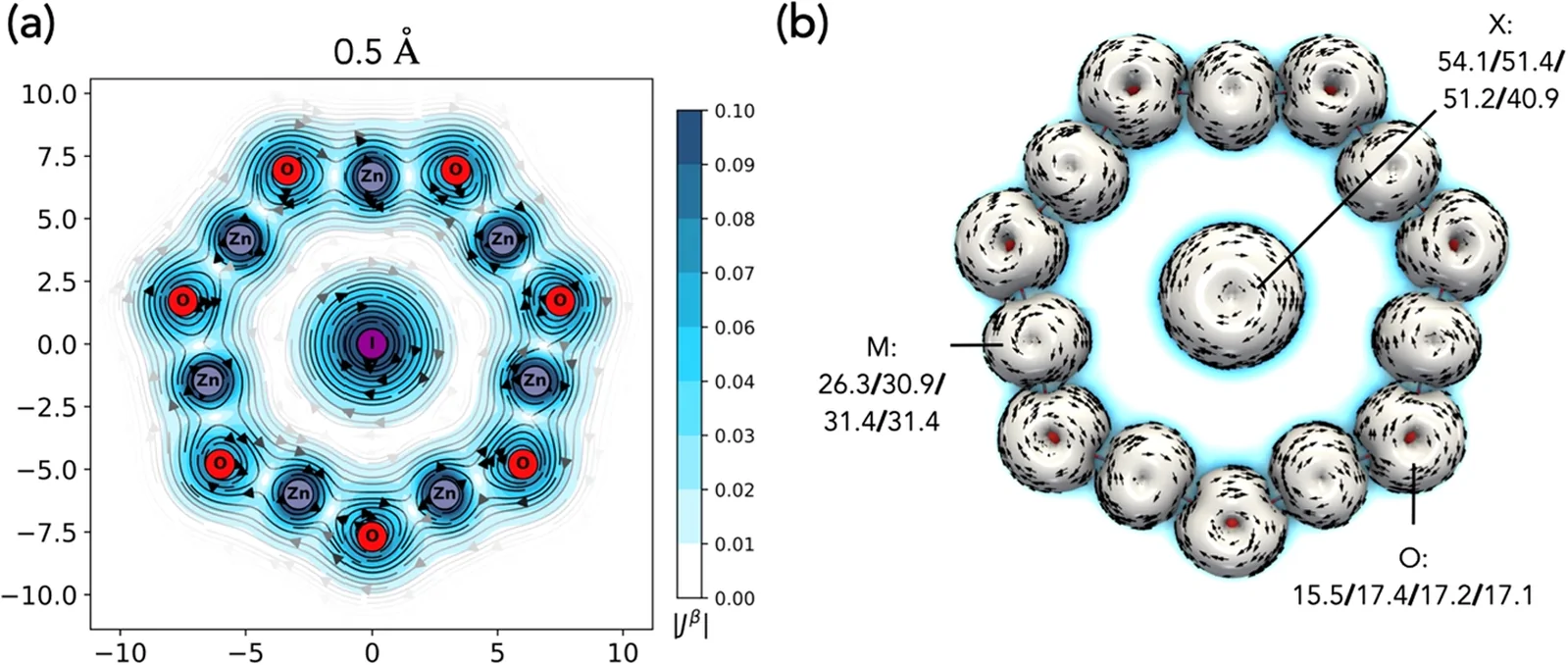
(a) MICD-vector plots of the *D*_7*h*_ I©Zn_7_O_7_^–^; (b) Ring-current strengths (RCSs, nA/T) of the X-, M-, and O-centered circuits. In each sequence, the values correspond to Br©Zn_7_O_7_^−^, I©Zn_7_O_7_^−^, At©Zn_7_O_7_^−^, and At©Cd_7_O_7_^−^, respectively.

The net ring current strength is negligible (≤0.1 nA/T), well below the values associated with aromatic systems and lower than that reported for the related phCl cluster Cl©Zn_6_O_6_^–^ (0.31 nA/T).^27^ The current density is dominated by diatropic vortices centered on individual atoms. The magnitude of these local currents follows the sequence X (40.9–54.1 nA/T) > M (26.3–31.4 nA/T) > O (15.5–17.4 nA/T), indicating that the magnetic response is atomic rather than cyclic.

The EDDB analysis provides a complementary picture of electron delocalization. The total population of delocalized electrons (5.86–5.92 |e|) indicates the presence of multicenter interactions within the M_7_O_7_ framework. However, the corresponding isosurface reveals that this delocalization is spatially fragmented: it is concentrated on oxygen atoms, connected by narrow regions at the metal sites, and only weakly involves the central halogen. The absence of a continuous delocalization pathway across the ring does not support aromatic behavior.

## Conclusion

This work identifies planar heptacoordinate halogen centers in four global-minimum structures (Br©Zn_7_O_7_^–^, I©Zn_7_O_7_^–^, At©Zn_7_O_7_^–^, and At©Cd_7_O_7_^–^), extending the highest planar coordination number reported for halogens to seven. The planar *D*_7*h*_ arrangement arises as a highly selective solution within the X©M_7_O_7_^–^ chemical space, requiring both a stable metal–oxide ring and compatibility between the halogen and the cavity size. The resulting systems are dynamically robust and electronically stable within a single-reference description. More broadly, the results indicate that planar halogen hypercoordination remains feasible up to *n* = 7 only within a narrow confinement window defined jointly by cavity size and framework rigidity.

Bonding analyses define a consistent picture. The M_7_O_7_ ring forms a covalent scaffold, while the central halogen remains electronically localized and interacts with the ring through electrostatic attraction distributed over the surrounding metal centers. This interaction pattern satisfies the IUPAC definition of coordination number and corresponds to heptacoordination without substantial direct covalent X–M bonding. Rather than introducing a new bonding paradigm, these systems show the persistence of an electrostatic confinement mechanism as the coordination number increases.

Magnetic and electron density analyses exclude aromatic contributions. Stability originates from electrostatic confinement within a rigid cavity rather than cyclic electron delocalization. Zn_7_O_7_ provides the confinement window required to stabilize Br, I, and At, whereas the larger and softer Cd_7_O_7_ cavity is compatible only with At. More broadly, the results indicate that planar halogen hypercoordination is retained to *n* = 7 only within a narrow confinement window jointly defined by cavity size and structural rigidity, rather than through the emergence of new bonding interactions.

This limitation is further reinforced by the instability of larger oxide rings. Within the Zn–O series, Zn_7_O_7_ is the largest ring considered here that retains a planar global-minimum structure, whereas Zn_8_O_8_ does not. This result prevents a direct extension of this specific host framework but does not exclude higher coordination numbers in other ring compositions. Thus, the upper coordination limit identified here is not only a consequence of cavity–halogen matching, but also of the structural stability of the host itself.

## Supplementary Material



## Notes

Optimized structures and relative energies of low-lying isomers; vibrational-frequency and electronic-structure data; Born–Oppenheimer molecular dynamics simulations; AdNDP, EDA–NOCV, EDDB, and IQA bonding analyses; vertical detachment energies; and Cartesian coordinates (PDF)

## References

[1] Kim J., Park E., Park J., Kim J., Seo W., Oh D., Lee J., Kim T. K. (2023). Do Planar Tetracoordinate Fluorine
Atoms Exist? Revisiting a Theoretical Prediction. J. Phys. Chem. A.

[2] Sarmah K., Kalita A. J., Guha A. K. (2024). Planar
tetracoordinate fluorine atom:
global minimum with viable possibility. Phys.
Chem. Chem. Phys..

[3] Li Y. X., Bai L. X., Guo J. C. (2024). FLi_4_X_4_
^–^ (X = Cl, Br, I): Superhalogen
Anions with Planar Tetracoordinate
Fluorine. Molecules.

[4] Sun R., Guan X. L., Yuan C., Wu Y. B. (2025). Star-like Cluster
S_6_Mg_5_
^2‑^ A Binary Dianion Global
Minimum Featuring a Planar Pentacoordinate Sulfur. Inorg. Chem..

[5] Yan M., Feng L. Y., Miao C. Q., Wang Y. J., Jin B. (2025). Alkali-Noble
Metal Synergy in F©Li_4_M_4_
^–^ (M = Ag, Au): A Novel Ionic Framework for Stabilizing Planar Tetracoordinate
Fluorine. J. Phys. Chem. A.

[6] Jin B., Yan M., Feng L. Y., Wang Z. R., Miao C. Q., Wang Y. J. (2025). Li_4_F_4_
^–^: Planar tetracoordinate fluorine
in a highly viable binary mono-anionic cluster. J. Chem. Phys..

[7] Cui L. J., Miao L. H., Orozco-Ic M., Li L., Pan S., Merino G., Cui Z. H. (2025). Planar Pentacoordinate Halogens. Angew. Chem..

[8] Monkhorst H. J. (1968). Activation
energy for interconversion of enantiomers containing an asymmetric
carbon atom without breaking bonds. Chem. Commun..

[9] Hoffmann R., Alder R. W., Wilcox C. F. (1970). Planar tetracoordinate
carbon. J. Am. Chem. Soc..

[10] Collins J. B., Dill J. D., Jemmis E. D., Apeloig Y., Schleyer P. v. R., Seeger R., Pople J. A. (1976). Stabilization
of planar tetracoordinate
carbon. J. Am. Chem. Soc..

[11] Li X., Wang L.-S., Boldyrev A. I., Simons J. (1999). Tetracoordinated Planar
Carbon in the Al_4_C^–^Anion. A Combined
Photoelectron Spectroscopy and ab Initio Study. J. Am. Chem. Soc..

[12] Pei Y., An W., Ito K., Schleyer P. V. R., Zeng X. C. (2008). Planar Pentacoordinate
Carbon in CAl_5_
^+^: A Global Minimum. J. Am. Chem. Soc..

[13] Leyva-Parra L., Diego L., Yañez O., Inostroza D., Barroso J., Vásquez-Espinal A., Merino G., Tiznado W. (2021). Planar Hexacoordinate Carbons: Half
Covalent, Half
Ionic. Angew. Chem., Int. Ed..

[14] Sacanamboy D. S., Gamero-Begazo P. L., Parco-Valencia K. E., Inostroza D., Ruiz L., Leyva-Parra L., Merino G., Tiznado W. (2024). Exploring
aromatic rings with planar tetracoordinate group 13–15 atoms. Chem. Commun..

[15] García-Argote W., Sacanamboy D. S., Vasquez-Espinal A., Inostroza D., Leyva-Parra L., Yañez O., Tiznado W. (2025). Doubly σ- and
π-aromatic planar pentacoordinate boron polyanions. Chem. Commun..

[16] Boldyrev A. I., Schleyer P. v. R. (1991). Ab initio prediction
of the structures and stabilities
of the hyperaluminum molecules: Al_3_O and square-planar
Al_4_O. J. Am. Chem. Soc..

[17] Das P., Chattaraj P. K. (2022). Structure
and Bonding in Planar Hypercoordinate Carbon
Compounds. Chemistry.

[18] Vassilev-Galindo V., Pan S., Donald K. J., Merino G. (2018). Planar pentacoordinate carbons. Nat. Rev. Chem..

[19] Yang L. M., Ganz E., Chen Z., Wang Z. X., Schleyer P. v. R. (2015). Four
Decades of the Chemistry of Planar Hypercoordinate Compounds. Angew. Chem., Int. Ed..

[20] Bian J. H., Jin B., Zhao X. F., Sun R., Yuan C., Zhou C. Y., Wu Y. B. (2021). Nitrogen versus
carbon in planar pentacoordinate environments supported
by Be_5_H_n_ rings. RSC Adv..

[21] Jin B., Guan X.-L., Yan M., Wang Y.-J., Wu Y.-B. (2023). Planar
hexacoordinate beryllium: Covalent bonding between s–block
metals. Chem. - Eur. J..

[22] García-Argote W., Tiznado W., Leyva-Parra L., Pino-Rios R. (2026). O©Li_5_F_5_
^2–^: A Global Minimum with a
Planar Pentacoordinate Oxygen. Inorg. Chem..

[23] Vásquez-Espinal A., Pino-Rios R. (2026). Comment on “Noble gas decorated planar tetracoordinate
oxygen” by K. Sarmah, F. Yashmin, A. Das, L.-X. Bai, J.-C.
Guo and A. K. Guha, Phys. Chem. Chem. Phys., 2025, 27, 10923. Phys. Chem. Chem. Phys..

[24] Sacanamboy D. S., Roman-Ventura V., Yañez O., García-Argote W., Leyva-Parra L., Tiznado W. (2026). Planar tetracoordinate nitrogen in
main-group cationic clusters. Phys. Chem. Chem.
Phys..

[25] Wang M.-H., Kalita A. J., Orozco-Ic M., Yan G.-R., Chen C., Yan B., Castillo-Toraya G., Tiznado W., Guha A. K., Pan S., Merino G., Cui Z.-H. (2023). Planar pentacoordinate s-block metals. Chem. Sci..

[26] Jin B., Yan M., Feng L. Y., Wang Y. J., Wu Y. B. (2025). OLi_4_H_3_
^–^: A planar tetracoordinate oxygen cluster
with dynamic structural fluxionality. J. Chem.
Phys..

[27] Cheng Y. X., Bai L. X., Martínez-Villarino F., Guo J. C., Merino G. (2026). Planar hexacoordinate chlorine. Chem. Sci..

[28] Bai L. X., Cheng Y. X., Martínez-Villarino F., Diego L., Guo J. C., Merino G. (2026). Ligand rigidity as a design principle
for planar pentacoordinate fluorine. Chem. Commun..

[29] Reber A. C., Khanna S. N., Hunjan J. S., Beltran M. R. (2007). Rings, towers, cages
of ZnO. Eur. Phys. J..

[30] Wang B., Nagase S., Zhao J., Wang G. (2007). Structural Growth Sequences
and Electronic Properties of Zinc Oxide Clusters (ZnO)­n (n = 2–18). J. Phys. Chem. C.

[31] Srinivasaraghavan R., Chandiramouli R., Jeyaprakash B. G., Seshadri S. (2013). Quantum chemical studies
on CdO nanoclusters stability. Spectrochim.
Acta, Part A.

[32] Yañez O., Báez-Grez R., Inostroza D., Rabanal-León W. A., Pino-Rios R., Garza J., Tiznado W. (2019). AUTOMATON: A Program
That Combines a Probabilistic Cellular Automata and a Genetic Algorithm
for Global Minimum Search of Clusters and Molecules. J. Chem. Theory Comput..

[33] Küchle W., Dolg M., Stoll H., Preuss H. (1991). Ab initio pseudopotentials
for Hg through Rn: I. Parameter sets and atomic calculations. Mol. Phys..

[34] Küchle W., Dolg M., Stoll H., Preuss H. (1994). Energy-adjusted pseudopotentials
for the actinides. Parameter sets and test calculations for thorium
and thorium monoxide. J. Chem. Phys..

[35] Fuentealba P., Preuss H., Stoll H., Von Szentpály L. (1982). A proper account
of core-polarization with pseudopotentials: single valence-electron
alkali compounds. Chem. Phys. Lett..

[36] Fuentealba P., von Szentpaly L., Preuss H., Stoll H. (1985). Pseudopotential calculations
for alkaline-earth atoms. J. Phys. B: At., Mol.
Opt. Phys..

[37] Bergner A., Dolg M., Küchle W., Stoll H., Preuß H. (1993). Ab initio
energy-adjusted pseudopotentials for elements of groups 13–17. Mol. Phys..

[38] Chai J.-D., Head-Gordon M. (2008). Systematic
optimization of long-range corrected hybrid
density functionals. J. Chem. Phys..

[39] Weigend F., Ahlrichs R. (2005). Balanced basis sets of split valence, triple zeta valence
and quadruple zeta valence quality for H to Rn: Design and assessment
of accuracy. Phys. Chem. Chem. Phys..

[40] Frisch, M. J. ; Trucks, G. W. ; Schlegel, H. B. ; Scuseria, G. E. ; Robb, M. A. ; Cheeseman, J. R. ; Scalmani, G. ; Barone, V. ; Petersson, G. A. ; Nakatsuji, H. ; Li, X. ; Caricato, M. ; Marenich, A. V. ; Bloino, J. ; Janesko, B. G. ; Gomperts, R. ; Mennucci, B. ; Hratchian, H. P. ; Ortiz, J. V. ; Izmaylov, A. F. ; Sonnenberg, J. L. ; Williams-Young, D. ; Ding, F. ; Lipparini, F. ; Egidi, F. ; Goings, J. ; Peng, B. ; Petrone, A. ; Henderson, T. ; Ranasinghe, D. ; Zakrzewski, V. G. ; Gao, J. ; Rega, N. ; Zheng, G. ; Liang, W. ; Hada, M. ; Ehara, M. ; Toyota, K. ; Fukuda, R. ; Hasegawa, J. ; Ishida, M. ; Nakajima, T. ; Honda, Y. ; Kitao, O. ; Nakai, H. ; Vreven, T. ; Throssell, K. ; Montgomery, J. A., Jr. ; Peralta, J. E. ; Ogliaro, F. ; Bearpark, M. J. ; Heyd, J. J. ; Brothers, E. N. ; Kudin, K. N. ; Staroverov, V. N. ; Keith, T. A. ; Kobayashi, R. ; Normand, J. ; Raghavachari, K. ; Rendell, A. P. ; Burant, J. C. ; Iyengar, S. S. ; Tomasi, J. ; Cossi, M. ; Millam, J. M. ; Klene, M. ; Adamo, C. ; Cammi, R. ; Ochterski, J. W. ; Martin, R. L. ; Morokuma, K. ; Farkas, O. ; Foresman, J. B. ; Fox, D. J. Gaussian 16, Revision C.02; Gaussian, Inc.: Wallingford, CT, 2016.

[41] Riplinger C., Neese F. (2013). An efficient and near linear scaling pair natural orbital based local
coupled cluster method. J. Chem. Phys..

[42] Riplinger C., Sandhoefer B., Hansen A., Neese F. (2013). Natural triple excitations
in local coupled cluster calculations with pair natural orbitals. J. Chem. Phys..

[43] Riplinger C., Pinski P., Becker U., Valeev E. F., Neese F. (2016). Sparse maps—A
systematic infrastructure for reduced-scaling electronic structure
methods. II. Linear scaling domain based pair natural orbital coupled
cluster theory. J. Chem. Phys..

[44] Truhlar D. G. (1998). Basis-set
extrapolation. Chem. Phys. Lett..

[45] Neese F., Hansen A., Liakos D. G. (2009). Efficient
and accurate approximations
to the local coupled cluster singles doubles method using a truncated
pair natural orbital basis. J. Chem. Phys..

[46] Neese F. (2025). Software Update:
The ORCA Program System—Version 6.0. WIREs Comput. Mol. Sci..

[47] Andrae D., Häußermann U., Dolg M., Stoll H., Preuß H. (1990). Energy-adjusted ab initio pseudopotentials for the
second and third row transition elements. Theor.
Chim. Acta.

[48] Köster, A. M. ; Geudtner, G. ; Alvarez-Ibarra, A. ; Calaminici, P. ; Casida, M. E. ; Carmona-Espindola, J. ; Dominguez, V. D. ; Flores-Moreno, R. ; Gamboa, G. U. ; Goursot, A. ; Heine, T. ; Ipatov, A. ; de la Lande, A. ; Janetzko, F. ; del Campo, J. M. ; Mejia-Rodriguez, D. ; Reveles, J. U. ; Vasquez-Perez, J. ; Vela, A. ; Zuniga-Gutierrez, B. ; Salahub, D. R. deMon2k, Version 6; The deMon Developers; Cinvestav, Mexico City, 2018.

[49] Geudtner G., Calaminici P., Carmona-Espíndola J., Del Campo J. M., Domínguez-Soria V. D., Moreno R. F., Gamboa G. U., Goursot A., Köster A. M., Reveles J. U., Mineva T., Vásquez-Pérez J. M., Vela A., Zúñinga-Gutierrez B., Salahub D. R. (2012). deMon2k. WIREs Comput. Mol. Sci..

[50] Wiberg K.
B. (1968). Application
of the Pople-Santry-Segal CNDO method to the cyclopropylcarbinyl and
cyclobutyl cation and to bicyclobutane. Tetrahedron.

[51] Reed A. E., Weinstock R. B., Weinhold F. (1985). Natural population analysis. J. Chem. Phys..

[52] Glendening E. D., Landis C. R., Weinhold F. (2019). NBO 7.0: New
vistas in localized
and delocalized chemical bonding theory. J.
Comput. Chem..

[53] Zubarev D. Yu., Boldyrev A. I. (2008). Developing paradigms of chemical bonding: Adaptive
natural density partitioning. Phys. Chem. Chem.
Phys..

[54] Lu T., Chen F. (2012). Multiwfn: A multifunctional wavefunction analyzer. J. Comput. Chem..

[55] Pendás A. M., Blanco M. A., Francisco E. (2004). Two-electron
integrations in the
quantum theory of atoms in molecules. J. Chem.
Phys..

[56] Blanco M. A., Martín Pendás A., Francisco E. (2005). Interacting
Quantum Atoms: A Correlated Energy Decomposition Scheme Based on the
Quantum Theory of Atoms in Molecules. J. Chem.
Theory Comput..

[57] Pendás A. M., Blanco M. A., Francisco E. (2007). Chemical fragments in real space:
Definitions, properties, and energetic decompositions. J. Comput. Chem..

[58] Adamo C., Barone V. (1999). Toward reliable density
functional methods without
adjustable parameters: The PBE0 model. J. Chem.
Phys..

[59] Grimme S., Antony J., Ehrlich S., Krieg H. (2010). A consistent and accurate
ab initio parametrization of density functional dispersion correction
(DFT-D) for the 94 elements H–Pu. J.
Chem. Phys..

[60] Keith, T. A. ; AIMAll (19.02.12) - TK Gristmill Software: Overland Park, KS, USA, 2019 (aim.tkgristmill.com).

[61] Mitoraj M. P., Michalak A., Ziegler T. (2009). A Combined
Charge and Energy Decomposition
Scheme for Bond Analysis. J. Chem. Theory Comput..

[62] Baerends E.
J., Aguirre N. F., Austin N. D., Autschbach J., Bickelhaupt F. M., Bulo R., Cappelli C., van Duin A. C. T., Egidi F., Fonseca Guerra C., Förster A., Franchini M., Goumans T. P. M., Heine T., Hellström M., Jacob C. R., Jensen L., Krykunov M., van Lenthe E., Michalak A., Mitoraj M. M., Neugebauer J., Nicu V. P., Philipsen P., Ramanantoanina H., Rüger R., Schreckenbach G., Stener M., Swart M., Thijssen J. M., Trnka T., Visscher L., Yakovlev A., van Gisbergen S. J. A. (2025). The Amsterdam Modeling Suite. J. Chem. Phys..

[63] Fliegl H., Taubert S., Lehtonen O., Sundholm D. (2011). The gauge including
magnetically induced current method. Phys. Chem.
Chem. Phys..

[64] Jusélius J., Sundholm D., Gauss J. (2004). Calculation of current densities
using gauge-including atomic orbitals. J. Chem.
Phys..

[65] Lazzeretti P., Malagoli M., Zanasi R. (1994). Computational approach to molecular
magnetic properties by continuous transformation of the origin of
the current density. Chem. Phys. Lett..

[66] Szczepanik D. W., Andrzejak M., Dominikowska J., Pawełek B., Krygowski T. M., Szatylowicz H., Solà M. (2017). The electron density of delocalized bonds (EDDB) applied
for quantifying aromaticity. Phys. Chem. Chem.
Phys..

[67] Szczepanik D. W., Andrzejak M., Dyduch K., Żak E., Makowski M., Mazur G., Mrozek J. (2014). A uniform approach
to the description of multicenter bonding. Phys.
Chem. Chem. Phys..

[68] de
Oliveira G., Martin J. M. L., De Proft F., Geerlings P. (1999). Electron affinities
of the first- and second-row atoms: Benchmark ab initio and density-functional
calculations. Phys. Rev. A.

[69] Kossmann S., Kirchner B., Neese F. (2007). Performance
of modern density functional
theory for the prediction of hyperfine structure: meta-GGA and double
hybrid functionals. Mol. Phys..

[70] Jensen K. P. (2008). Bioinorganic
chemistry modeled with the TPSSh density functional. Inorg. Chem..

[71] Pyykkö P. (2015). Additive Covalent
Radii for Single-, Double-, and Triple-Bonded Molecules and Tetrahedrally
Bonded Crystals: A Summary. J. Phys. Chem. A.

[72] Cederbaum L. S. (1975). One-body
Green’s function for atoms and molecules: theory and application. J. Phys. B: At., Mol. Opt. Phys..

[73] Ortiz J. V. (1988). Electron
binding energies of anionic alkali metal atoms from partial fourth
order electron propagator theory calculations. J. Chem. Phys..

[74] Ortiz J. (1999). Toward an
exact one-electron picture of chemical bonding. Adv. Quantum Chem..

