# Supplementary material for: Planar Heptacoordinate Halogens
Source: ACS Phys Chem Au. 2026 Aug 28;6(5):1331–9. doi: 10.1021/acsphyschemau.6c00103 (PMC13614124; doi:10.1021/acsphyschemau.6c00103)
Supplement: Supplementary file 1 [file pg6c00103_si_001.pdf]

Supplementary Information

Planar Heptacoordinate Halogens

Williams García-Argote,[a,b], Alejandro Vasquez-Espinal,[c] Lina Ruiz,[d,*] Luis Leyva-Parra,[a,*] Gabriel Merino,[e,*] and William Tiznado.[a,*]

[a]*Centro de Investigación para el Diseño de Materiales (CEDEM), Facultad de Ciencias Exactas, Departamento de Ciencias Químicas, Universidad Andres Bello, Avenida República 275, Santiago 8370146, Chile*

[b]*Centro de Modelación Ambiental y Dinámica de Sistemas (CEMADIS), Facultad de Ingeniería y Negocios, Universidad de Las Américas, Santiago 7500975, Chile*

[c]*Química y Farmacia, Facultad de Ciencias de la Salud, Universidad Arturo Prat, Casilla 121, Iquique 1100000, Chile*

[d]*Instituto de Ciencias Biomédicas, Facultad de Ciencias de la Salud, Universidad Autónoma de Chile, Santiago de Chile 8370146, Chile*

[e]*Departamento de Física Aplicada, Centro de Investigación y de Estudios Avanzados, Unidad Mérida, Km 6 Antigua Carretera a Progreso. Mérida 97310, Mexico*

E-mail: lina.ruiz@uautonoma.cl, luis.leyva@unab.cl, gmerino@cinvestav.mx, wtiznado@unab.cl

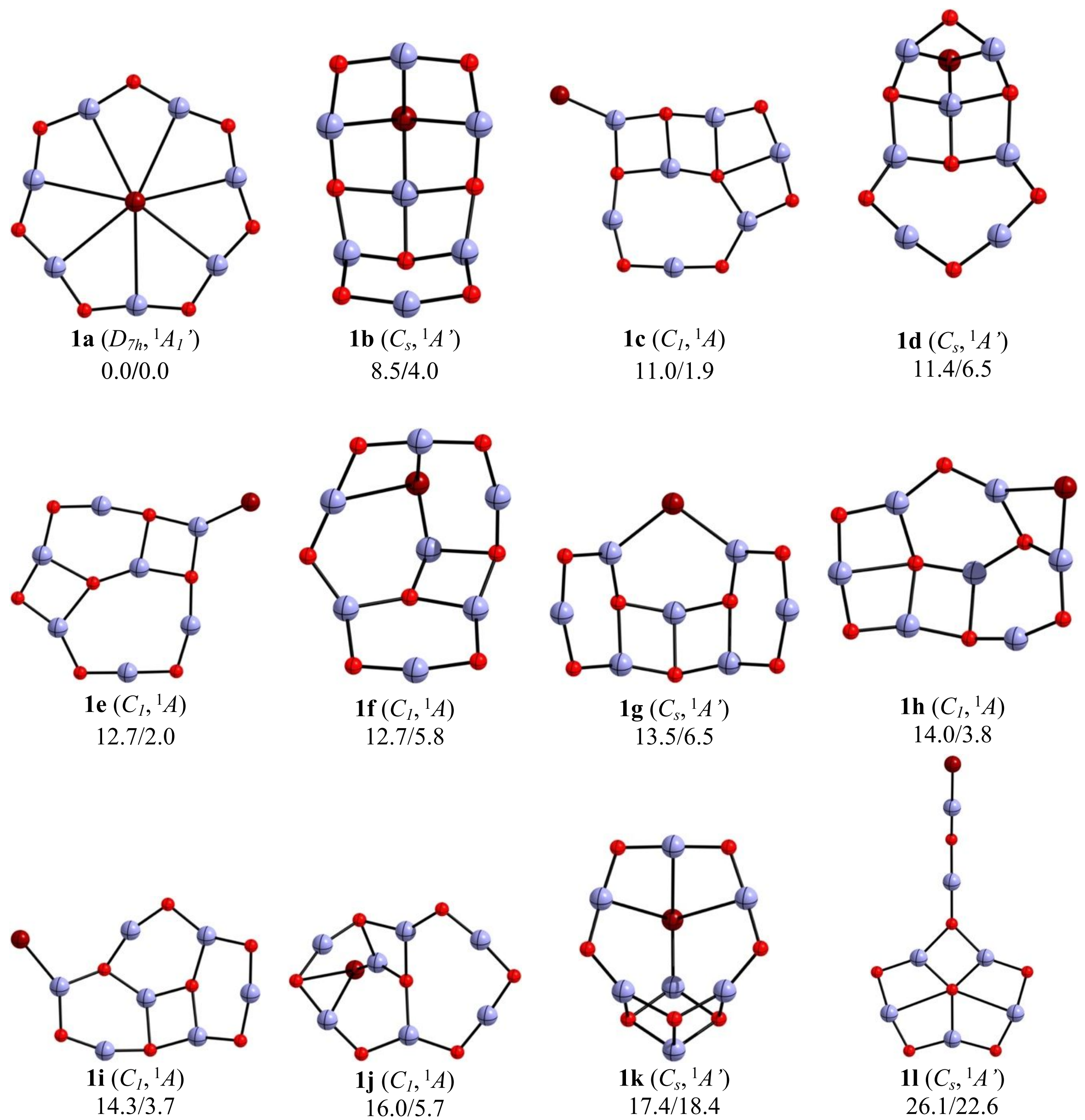

1a (D_{7h}, $^1A_1'$) 0.0/0.0

1b (C_s, $^1A'$) 8.5/4.0

1c (C_1, ^1A) 11.0/1.9

1d (C_s, $^1A'$) 11.4/6.5

1e (C_1, ^1A) 12.7/2.0

1f (C_1, ^1A) 12.7/5.8

1g (C_s, $^1A'$) 13.5/6.5

1h (C_1, ^1A) 14.0/3.8

1i (C_1, ^1A) 14.3/3.7

1j (C_1, ^1A) 16.0/5.7

1k (C_s, $^1A'$) 17.4/18.4

1l (C_s, $^1A'$) 26.1/22.6

Figure S1. ωB97X-D/def2-TZVPP geometries of the low-lying isomers of $BrZn_7O_7^-$. The first and second relative energies (kcal mol^{-1}) correspond to DLPNO-CCSD(T)/CBS and ωB97X-D/def2-TZVPP, respectively, and include ZPE corrections. Point groups and spectroscopic states are included.

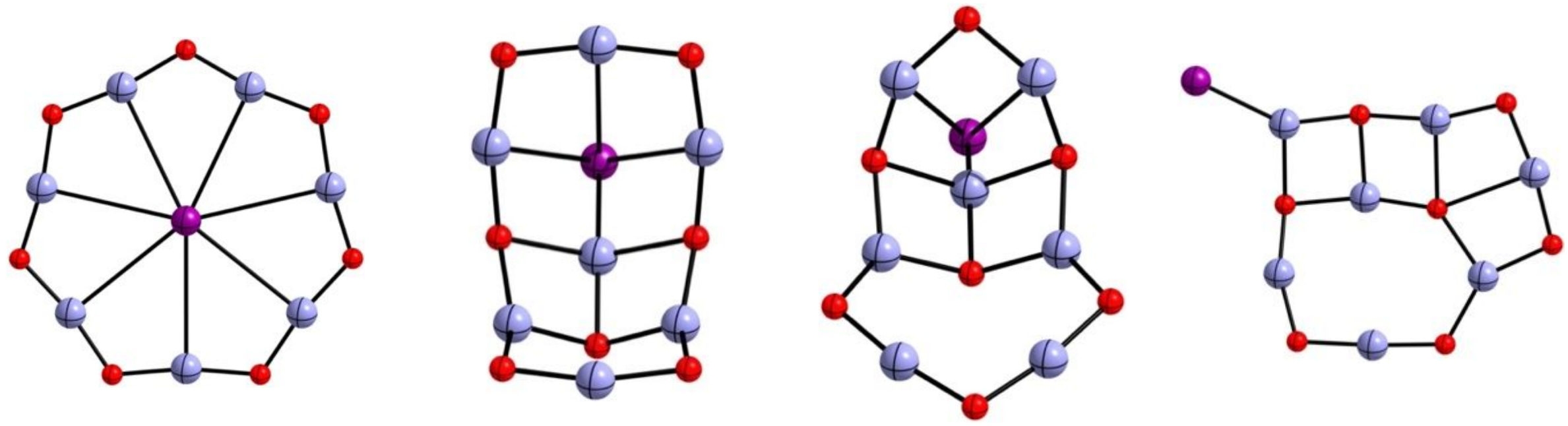

2a (D_{7h}, $^1A_1'$) 0.0/0.0

2b (C_s, $^1A'$) 9.9/6.8

2c (C_s, $^1A'$) 14.9/8.3

2d (C_1, ^1A) 15.0/3.9

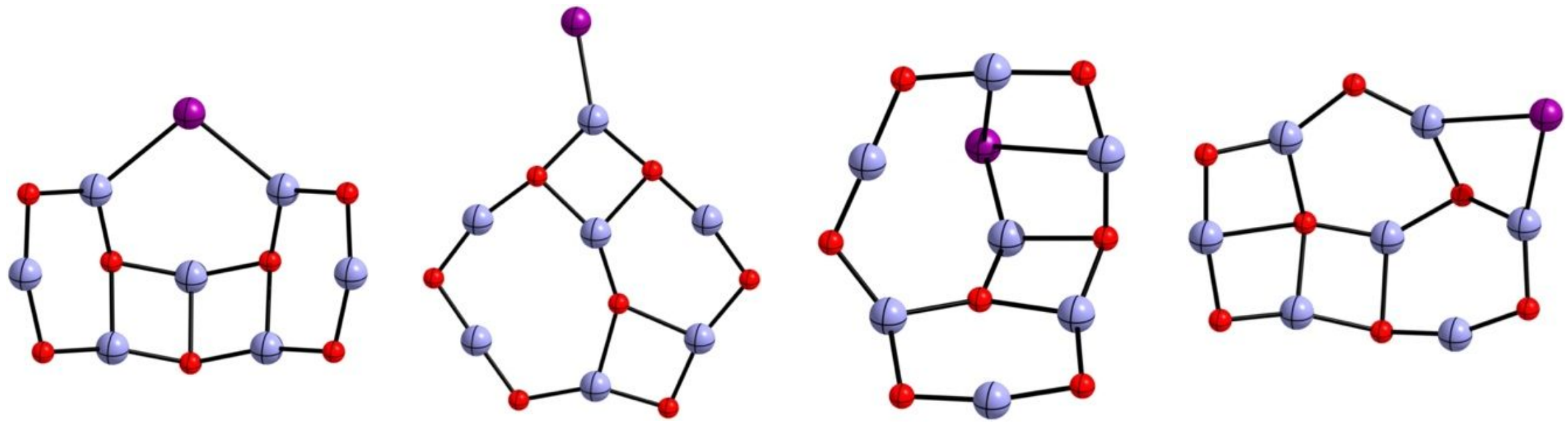

2e (C_s, $^1A'$) 16.3/8.0

2f (C_1, ^1A) 17.3/4.2

2g (C_1, ^1A) 17.6/7.9

2h (C_1, ^1A) 18.6/6.0

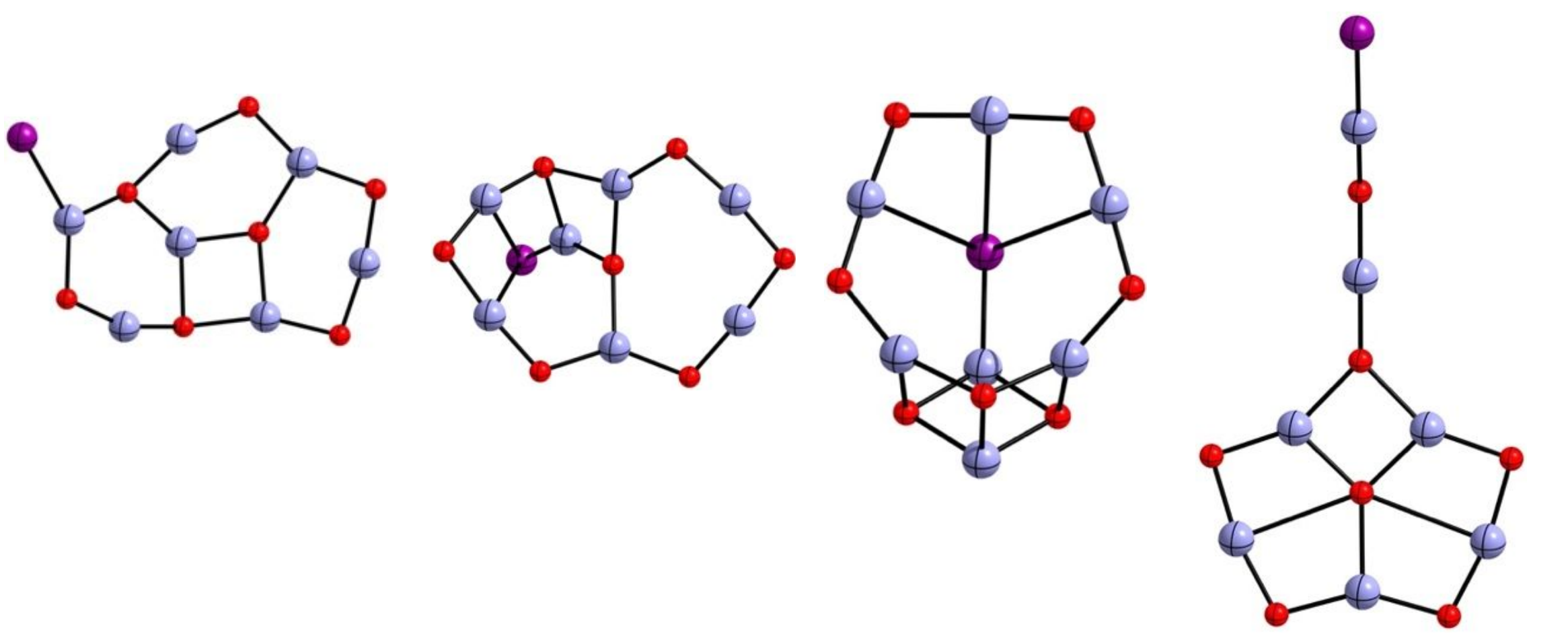

2i (C_1, ^1A) 18.9/5.7

2j (C_1, ^1A) 19.8/7.8

2k (C_s, $^1A'$) 21.7/19.9

2l (C_1, ^1A) 31.1/25.1

Figure S2. ωB97X-D/def2-TZVPP geometries of the low-lying isomers of $IZn_7O_7^-$. The first and second relative energies (kcal mol^{-1}) correspond to DLPNO-CCSD(T)/CBS and ωB97X-D/def2-TZVPP, respectively, and include ZPE corrections. Point groups and spectroscopic states are included.

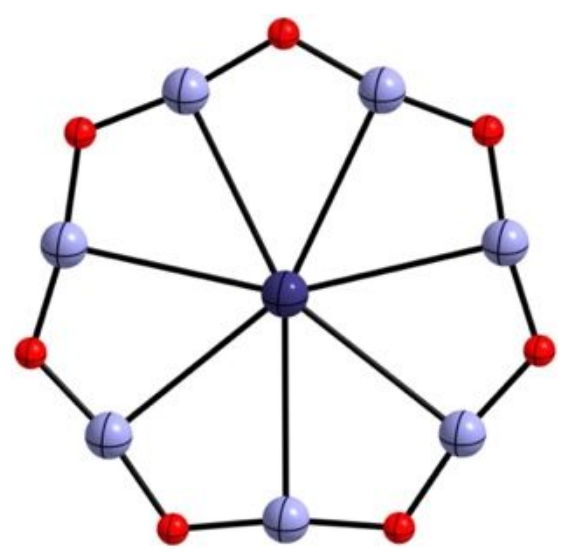

3a (D_{7h}, $^{1}A_{1}'$)
0.0/0.0/0.0

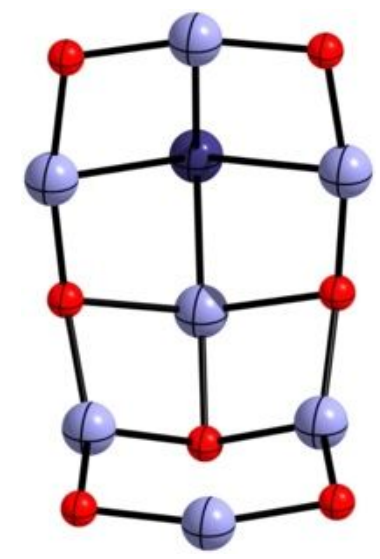

3b (C_s, $^{1}A'$)
8.8/7.4/13.8

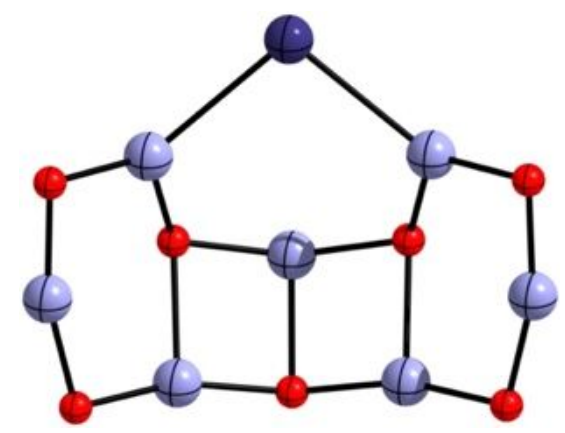

3c (C_s, $^{1}A'$)
13.2/8.7/16.3

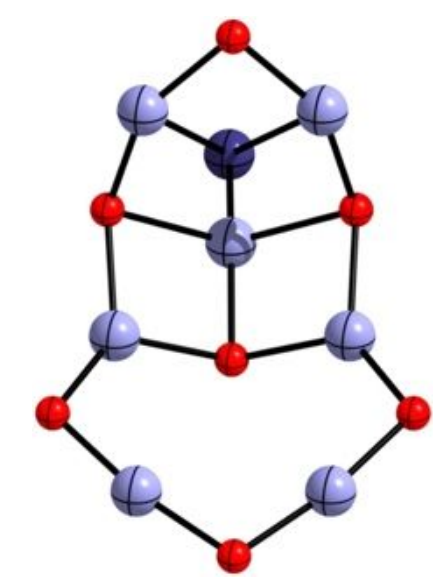

3d (C_s, $^{1}A'$)
13.5/8.9/15.9

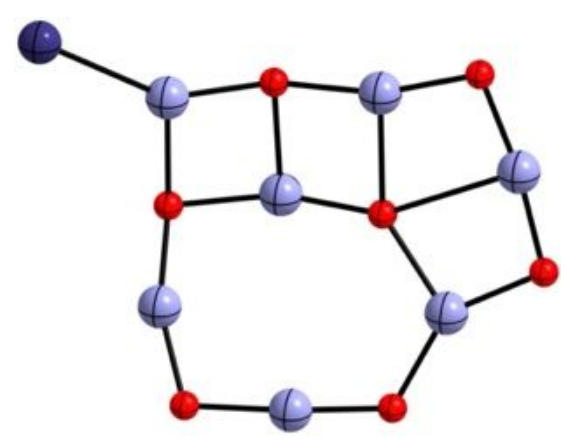

3e (C_1, ^{1}A)
13.8/5.0

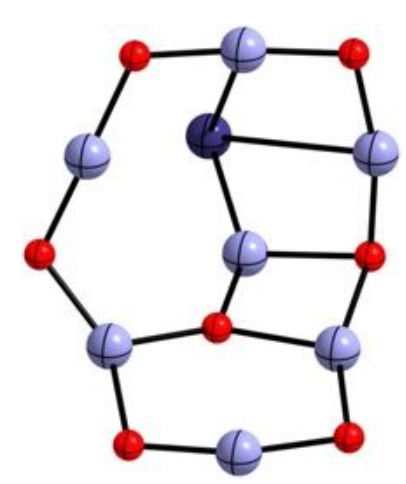

3f (C_1, ^{1}A)
14.4/8.5

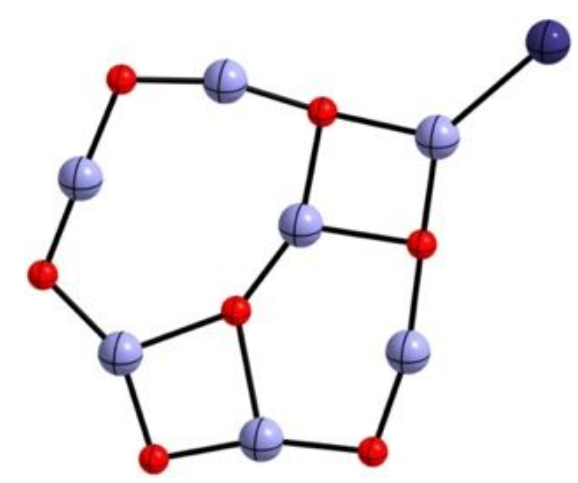

3g (C_1, ^{1}A)
16.0/5.2

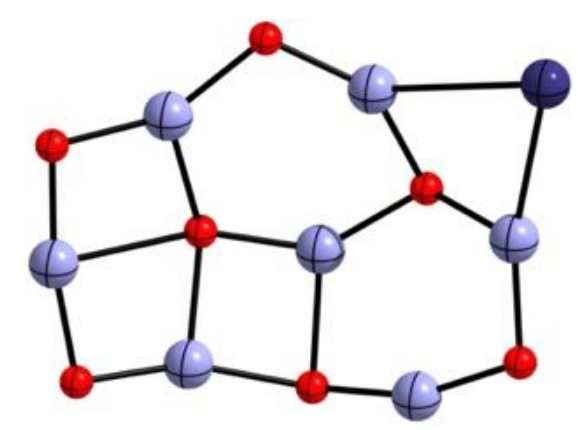

3h (C_1, ^{1}A)
16.6/6.7

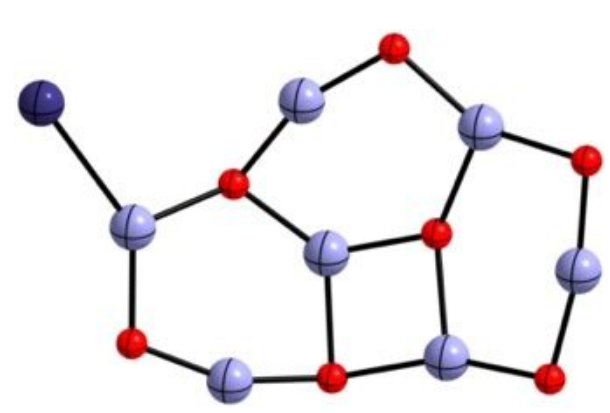

3i (C_1, ^{1}A)
16.7/6.6

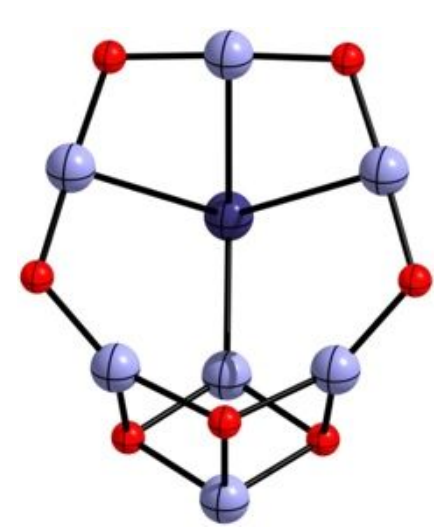

3j (C_s, $^{1}A'$)
17.7/20.2

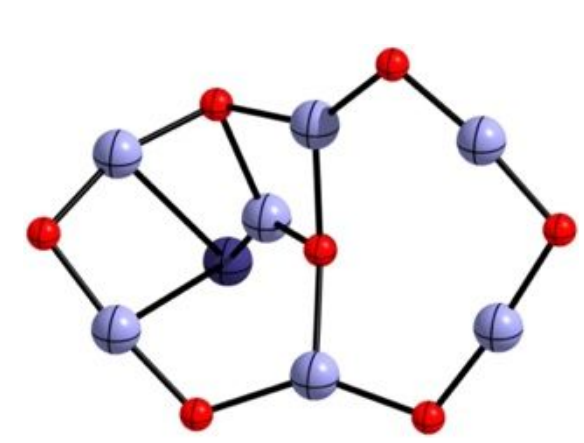

3k (C_1, ^{1}A)
18.0/8.6

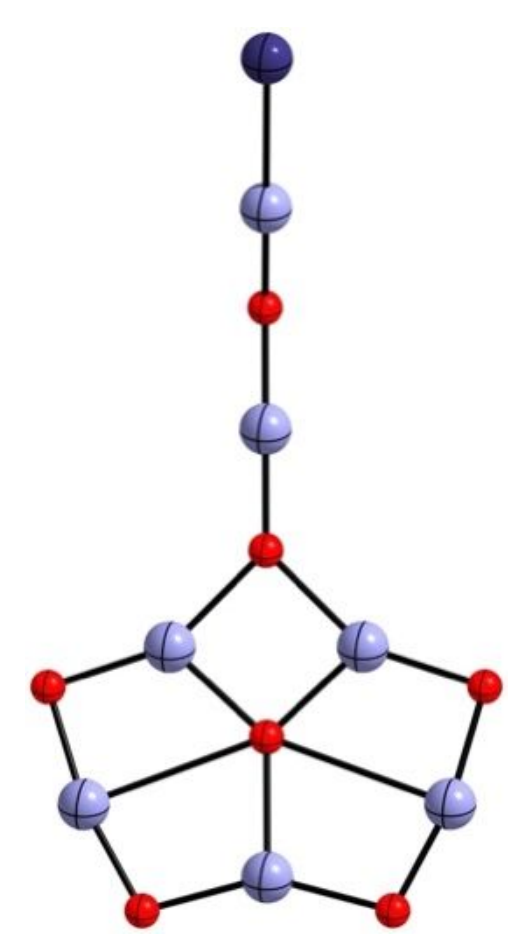

3l (C_s, $^{1}A'$)
28.3/26.0

Figure S3. ωB97X-D/def2-TZVPP geometries of the low-lying isomers of $AtZn_7O_7^-$. Relative energies (kcal mol^{-1}) are reported in order DLPNO-CCSD(T)/CBS, ωB97X-D/def2-TZVPP, and ωB97X-D3/TZ2P-ZORA, where available, and include ZPE corrections. Point groups and spectroscopic states are included.

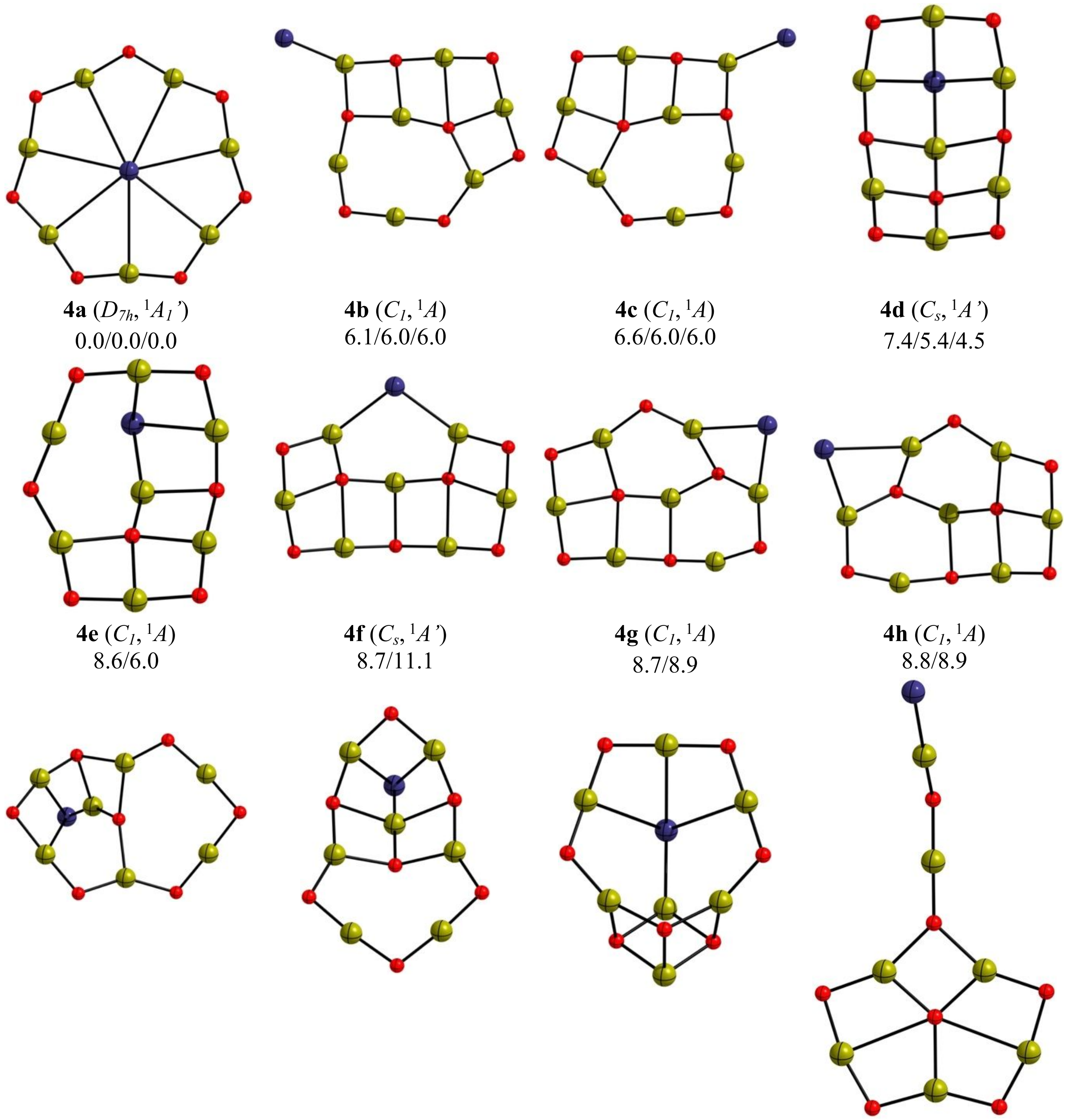

4a (D_{7h}, $^1A_1'$) 0.0/0.0/0.0

4b (C_1, ^1A) 6.1/6.0/6.0

4c (C_1, ^1A) 6.6/6.0/6.0

4d (C_s, $^1A'$) 7.4/5.4/4.5

4e (C_1, ^1A) 8.6/6.0

4f (C_s, $^1A'$) 8.7/11.1

4g (C_1, ^1A) 8.7/8.9

4h (C_1, ^1A) 8.8/8.9

4i (C_1, ^1A) 13.1/10.1

4j (C_s, $^1A'$) 14.6/12.0

4k (C_s, $^1A'$) 23.8/23.9

4l (C_1, ^1A) 28.2/29.2

Figure S4. ωB97X-D/def2-TZVPP geometries of the low-lying isomers of $AtCd_7O_7^-$. Relative energies (kcal mol^{-1}) are reported in order DLPNO-CCSD(T)/CBS, ωB97X-D/def2-TZVPP, and ωB97X-D3/TZ2P-ZORA, where available, and include ZPE corrections. Point groups and spectroscopic states are included.

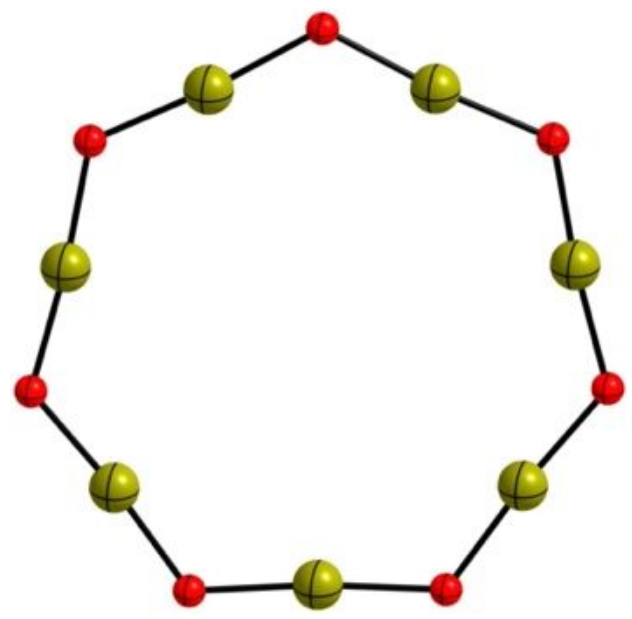

5a (D_{7h}, $^1A_1'$)
0.0/0.0

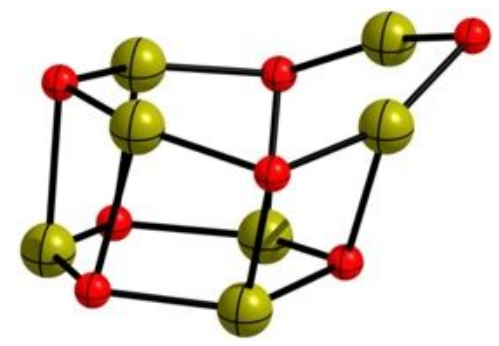

5b (C_1, ^1A)
3.2/1.1

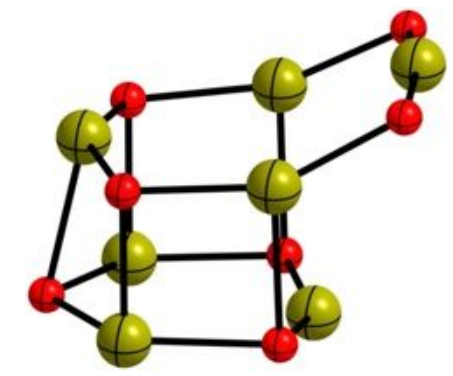

5c (C_s, $^1A'$)
7.8/3.3

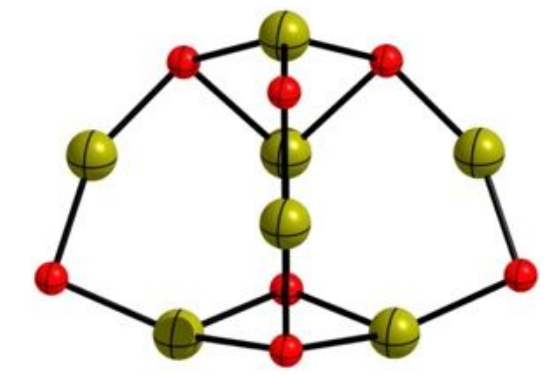

5d (C_s, $^1A'$)
5.2/4.3

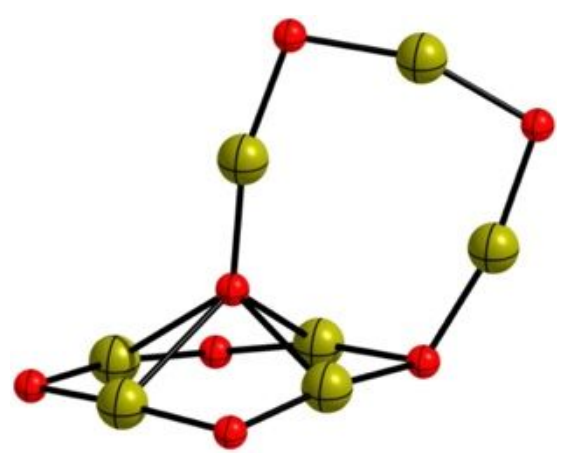

5e (C_s, $^1A'$)
16.7/9.8

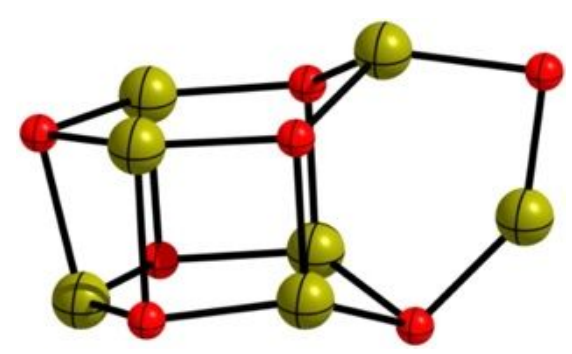

5f (C_s, $^1A'$)
19.2/12.1

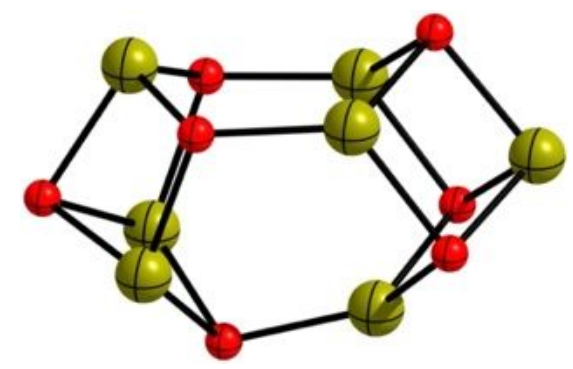

5g (C_{3v}, $^1A_1$)
24.5/17.2

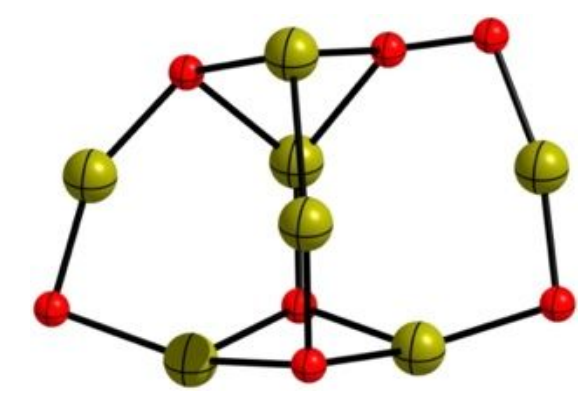

5h (C_1, ^1A)
60.9/52.0

Figure S5. ωB97X-D/def2-TZVPP geometries of the low-lying isomers of Cd_7O_7. Relative energies (kcal mol^{-1}) are reported in the order DLPNO-CCSD(T)/CBS, ωB97X-D/def2-TZVPP, and ωB97X-D3/TZ2P-ZORA, where available, and include ZPE corrections. Point groups and spectroscopic states are included.

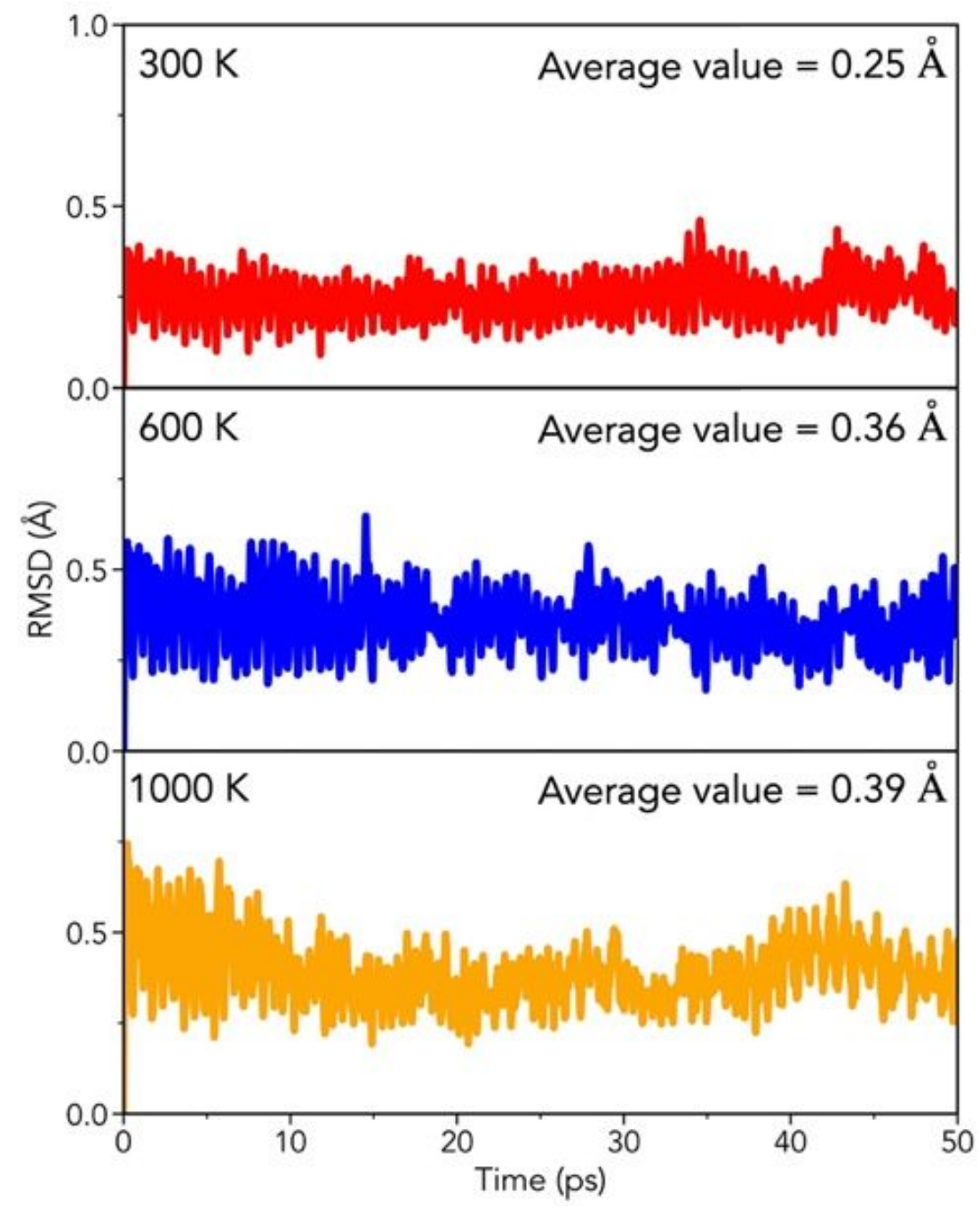

Figure S6. Root-mean-square deviation (RMSD) as a function of time during 50 ps Born-Oppenheimer molecular dynamics simulations of D_{7h}–I©$Zn_7O_7^-$ at 300, 600, and 1000 K.

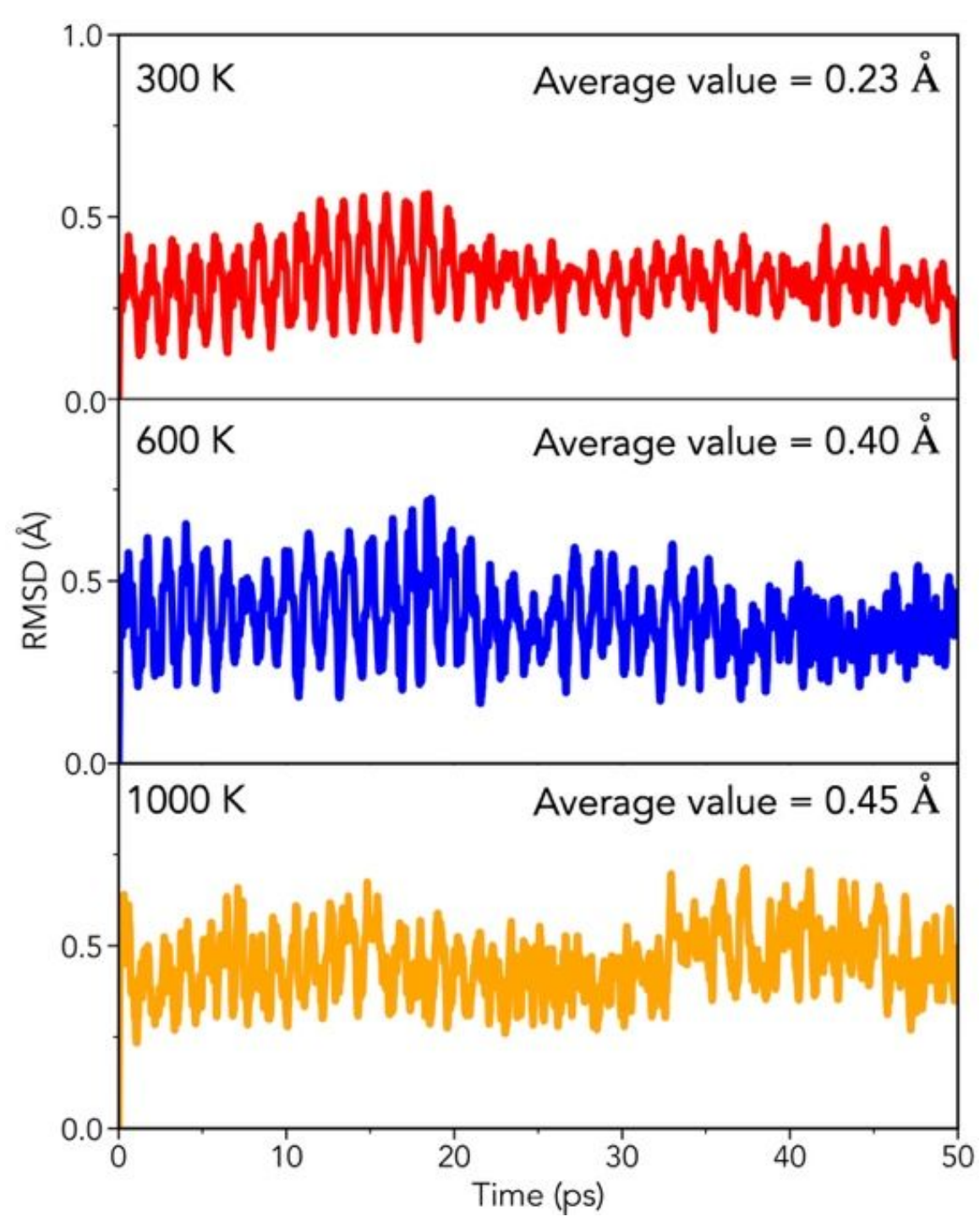

Figure S7. Root-mean-square deviation (RMSD) as a function of time during 50 ps Born-Oppenheimer molecular dynamics simulations of D_{7h}–At©$Cd_7O_7^-$ at 300, 600, and 1000 K.

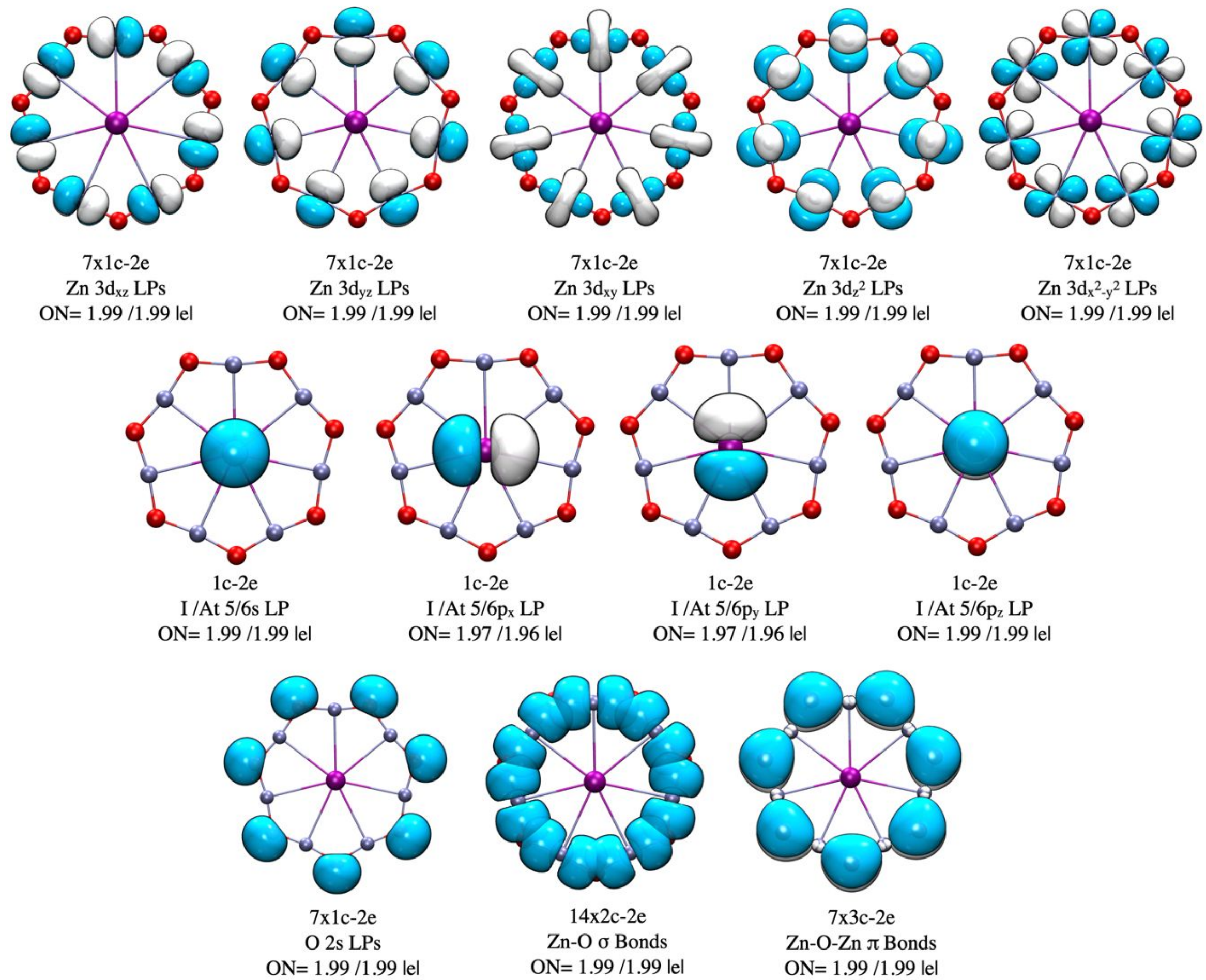

Figure S8. Adaptive Natural Density Partitioning (AdNDP) analysis of I©$Zn_7O_7^-$ and At©$Zn_7O_7^-$ at the ωB97X-D/def2-TZVPP level. Occupation numbers (ONs) are given in |e|.

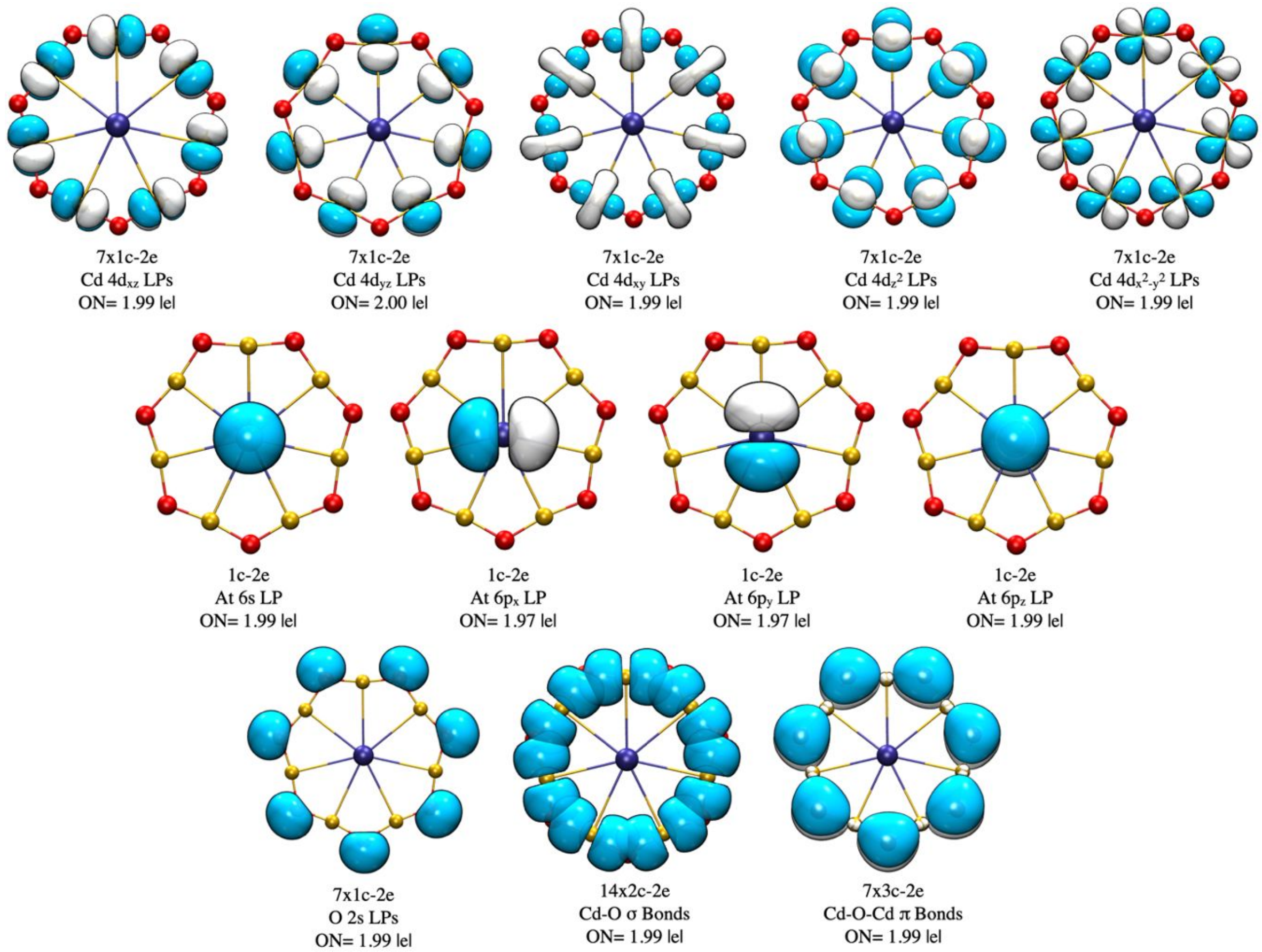

Figure S9. Adaptive Natural Density Partitioning (AdNDP) analysis of At©$Cd_7O_7^-$ at the ωB97X-D/def2-TZVPP level. Occupation numbers (ONs) are given in |e|.

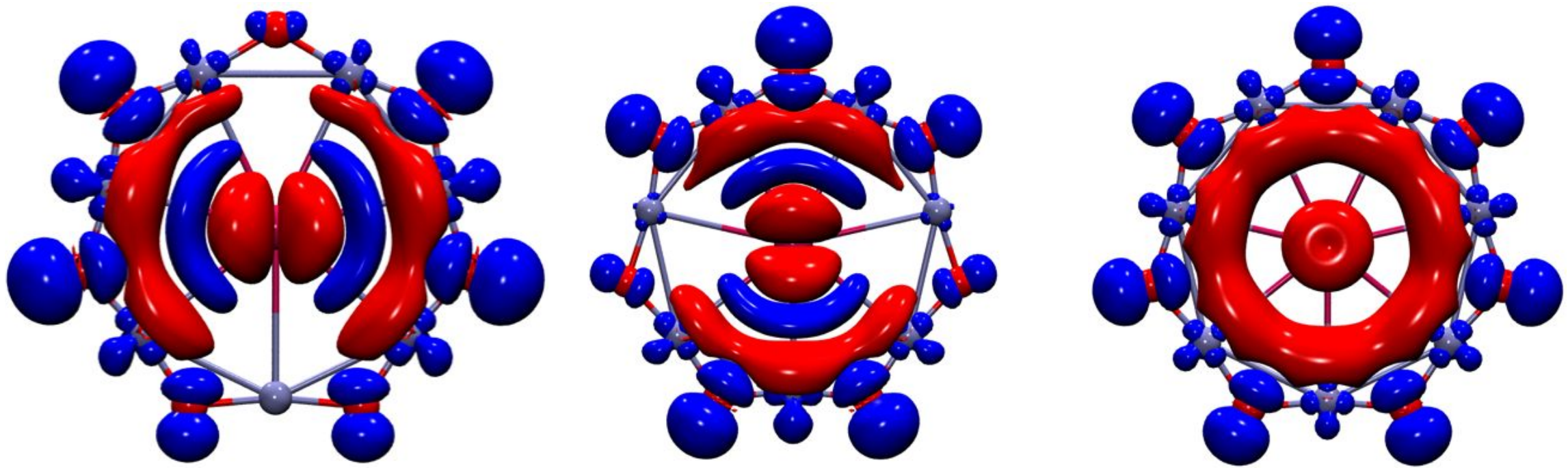

$\Delta E_{orb(1)} = -6.7$ $\Delta E_{orb(2)} = -6.7$ $\Delta E_{orb(3)} = -2.0$

Figure S10. Deformation density ($\Delta\rho$) plots from the EDA-NOCV analysis of Br©$Zn_7O_7^-$. The isosurfaces are shown at 0.0002 a.u. Charge flows from red (donor) to blue (acceptor). Energy values are given in kcal mol^{-1}.

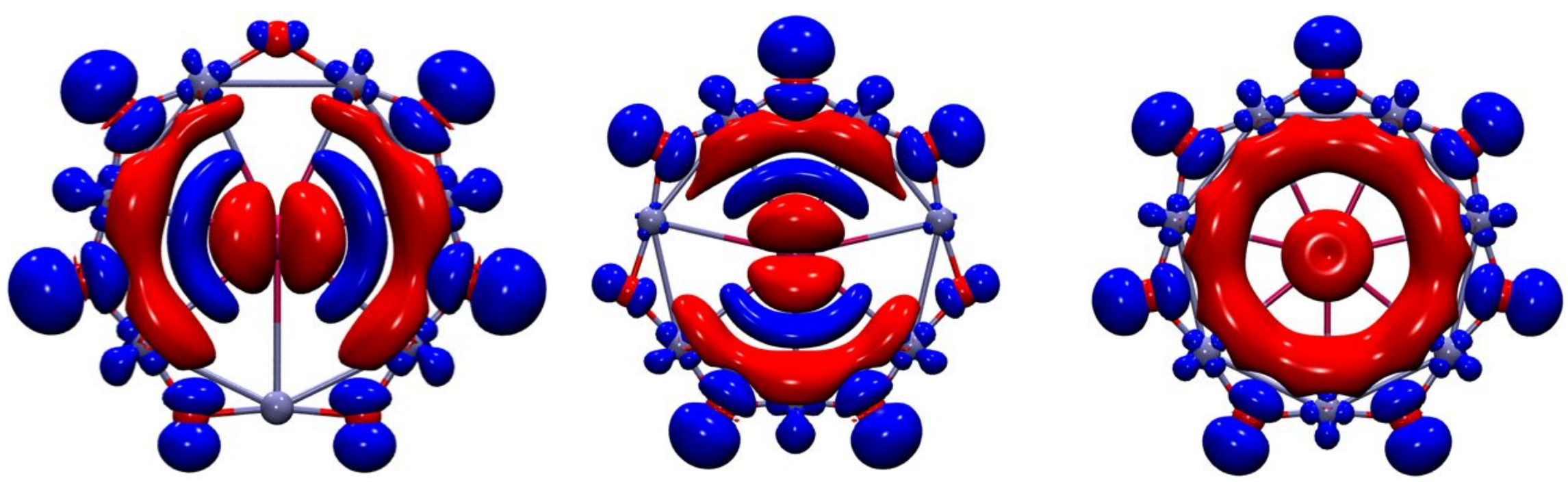

(a) $\Delta E_{orb(1)} = -7.7$ $\Delta E_{orb(2)} = -7.7$ $\Delta E_{orb(3)} = -1.6$

(b) $\Delta E_{orb(1)} = -8.6$ $\Delta E_{orb(2)} = -8.6$ $\Delta E_{orb(3)} = -1.1$

Figure S11. Deformation density ($\Delta\rho$) plots from the EDA-NOCV analysis of (a) I©$Zn_7O_7^-$ and (b) At©$Zn_7O_7^-$. The isosurfaces are shown at 0.0002 a.u. Charge flows from red (donor) to blue (acceptor). Energy values are given in kcal mol^{-1}.

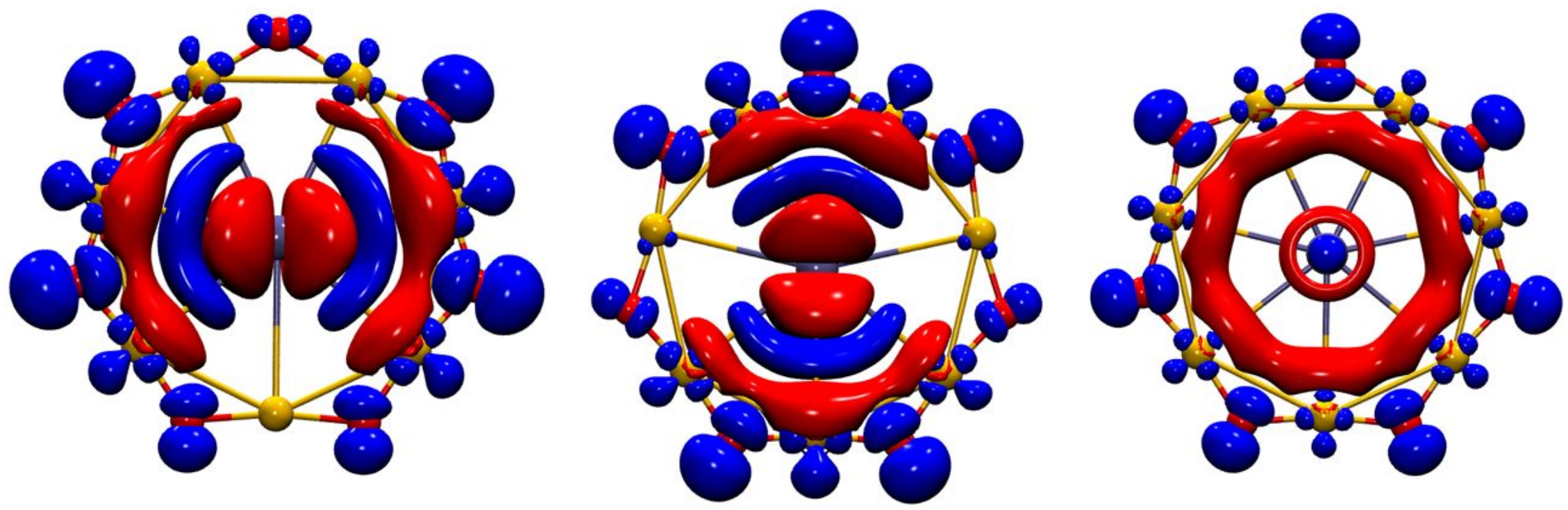

$\Delta E_{orb(1)} = -6.7$ $\Delta E_{orb(2)} = -6.7$ $\Delta E_{orb(3)} = -1.1$

Figure S12. Deformation density (Δρ) plots from the EDA-NOCV analysis of At©$Cd_7O_7^-$. The isosurfaces are shown at 0.0002 a.u. Charge flows from red (donor) to blue (acceptor). Energy values are given in kcal mol^{-1}.

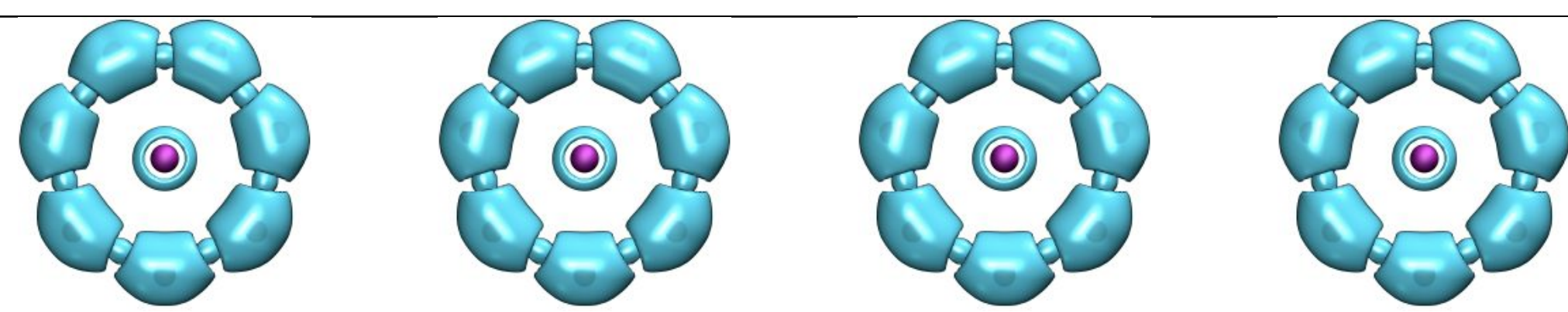

Br©$Zn_7O_7^-$	I©$Zn_7O_7^-$	At©$Zn_7O_7^-$	At©$Cd_7O_7^-$
5.87 |e|	5.86 |e|	5.92 |e|	7.06 |e|

Figure S13. (a) Isosurfaces of the $EDDB_G$ function at an isovalue of ±0.002 a.u; (b) corresponding populations of delocalized electrons for the D_{7h}–X©$M_7O_7^-$ systems at the ωB97X-D/def2-TZVPP level.

Table S1. Lowest vibrational frequencies (ν_{min}, cm^{-1}) and numbers of imaginary frequencies (NIMG) for D_{7h}-X©$M_7E_7^-$ systems (X = F-At; M = Zn-Hg; E = O-Po), computed at the ωB97X-D/def2-TZVPP level.

**System**	ν_{min}	**NIMG**	**System**	ν_{min}	**NIMG**	**System**	ν_{min}	**NIMG**
F©$Zn_7O_7^-$	120i	6	F©$Cd_7O_7^-$	102i	8	F©$Hg_7O_7^-$	87i	6
F©$Zn_7S_7^-$	115i	8	F©$Cd_7S_7^-$	99i	8	F©$Hg_7S_7^-$	97i	8
F©$Zn_7Se_7^-$	101i	8	F©$Cd_7Se_7^-$	99i	8	F©$Hg_7Se_7^-$	103i	8
F©$Zn_7Te_7^-$	108i	8	F©$Cd_7Te_7^-$	96i	8	F©$Hg_7Te_7^-$	80i	8
F©$Zn_7Po_7^-$	101i	8	F©$Cd_7Po_7^-$	99i	10	F©$Hg_7Po_7^-$	19i	6
Cl©$Zn_7O_7^-$	13i	4	Cl©$Cd_7O_7^-$	57i	6	Cl©$Hg_7O_7^-$	49i	6
Cl©$Zn_7S_7^-$	85i	8	Cl©$Cd_7S_7^-$	66i	8	Cl©$Hg_7S_7^-$	46i	6
Cl©$Zn_7Se_7^-$	88i	8	Cl©$Cd_7Se_7^-$	83i	8	Cl©$Hg_7Se_7^-$	63i	8
Cl©$Zn_7Te_7^-$	95i	10	Cl©$Cd_7Te_7^-$	65i	8	Cl©$Hg_7Te_7^-$	67i	6
Cl©$Zn_7Po_7^-$	95i	10	Cl©$Cd_7Po_7^-$	70i	10	Cl©$Hg_7Po_7^-$	66i	8
**Br©$Zn_7O_7^-$**	**10**	0	**Br©$Cd_7O_7^-$**	13i	2	**Br©$Hg_7O_7^-$**	39i	6
Br©$Zn_7S_7^-$	38i	8	Br©$Cd_7S_7^-$	44i	8	Br©$Hg_7S_7^-$	42i	8
Br©$Zn_7Se_7^-$	72i	10	Br©$Cd_7Se_7^-$	50i	8	Br©$Hg_7Se_7^-$	34i	8
Br©$Zn_7Te_7^-$	55i	8	Br©$Cd_7Te_7^-$	55i	10	Br©$Hg_7Te_7^-$	41i	8
Br©$Zn_7Po_7^-$	56i	10	Br©$Cd_7Po_7^-$	50i	10	Br©$Hg_7Po_7^-$	43i	8
**I©$Zn_7O_7^-$**	**15**	0	**I©$Cd_7O_7^-$**	5.2i	2	**I©$Hg_7O_7^-$**	27i	4
I©$Zn_7S_7^-$	36i	8	I©$Cd_7S_7^-$	35i	8	I©$Hg_7S_7^-$	39i	4
I©$Zn_7Se_7^-$	27i	8	I©$Cd_7Se_7^-$	25i	8	I©$Hg_7Se_7^-$	30i	8
I©$Zn_7Te_7^-$	37i	8	I©$Cd_7Te_7^-$	21i	8	I©$Hg_7Te_7^-$	20i	6
I©$Zn_7Po_7^-$	41i	8	I©$Cd_7Po_7^-$	41i	10	I©$Hg_7Po_7^-$	31i	8
**At©$Zn_7O_7^-$**	**4**	**0**	**At©$Cd_7O_7^-$**	**6**	**0**	**At©$Hg_7O_7^-$**	20i	4
At©$Zn_7S_7^-$	34i	4	At©$Cd_7S_7^-$	35i	4	At©$Hg_7S_7^-$	39i	4
At©$Zn_7Se_7^-$	25i	6	At©$Cd_7Se_7^-$	23i	8	At©$Hg_7Se_7^-$	27i	6
At©$Zn_7Te_7^-$	25i	8	At©$Cd_7Te_7^-$	36i	10	At©$Hg_7Te_7^-$	25i	8
At©$Zn_7Po_7^-$	26i	8	At©$Cd_7Po_7^-$	20i	8	At©$Hg_7Po_7^-$	18i	8

Table S2. Lowest vibrational frequencies (ν_{min}, cm^{-1}) and numbers of imaginary frequencies (NIMG) for D_{7h}-X©$A_7E_7^-$ systems (X = F-At; A = Be-Ba; E = O-Po), computed at the ωB97X-D/def2-TZVPP level.

System	ν_{min}	NIMG	System	ν_{min}	NIMG	System	ν_{min}	NIMG	System	ν_{min}	NIMG
F©$Be_7O_7^-$	160i	4	F©$Mg_7O_7^-$	133i	8	F©$Ca_7O_7^-$	112i	10	F©$Sr_7O_7^-$	94i	8
F©$Be_7S_7^-$	159i	8	F©$Mg_7S_7^-$	116i	10	F©$Ca_7S_7^-$	99i	10	F©$Sr_7S_7^-$	----	-----
F©$Be_7Se_7^-$	161i	10	F©$Mg_7Se_7^-$	134i	10	F©$Ca_7Se_7^-$	96i	10	F©$Sr_7Se_7^-$	74i	10
F©$Be_7Te_7^-$	149i	10	F©$Mg_7Te_7^-$	75i	8	F©$Ca_7Te_7^-$	95i	10	F©$Sr_7Te_7^-$	72i	10
F©$Be_7Po_7^-$	150i	10	F©$Mg_7Po_7^-$	130i	10	F©$Ca_7Po_7^-$	95i	10	F©$Sr_7Po_7^-$	71i	10
**Cl©$Be_7O_7^-$**	**33**	0	**Cl©$Mg_7O_7^-$**	65i	4	**Cl©$Ca_7O_7^-$**	85i	10	**Cl©$Sr_7O_7^-$**	72i	11
Cl©$Be_7S_7^-$	116i	8	Cl©$Mg_7S_7^-$	116i	8	Cl©$Ca_7S_7^-$	80i	10	Cl©$Sr_7S_7^-$	----	----
Cl©$Be_7Se_7^-$	60i	8	Cl©$Mg_7Se_7^-$	96i	10	Cl©$Ca_7Se_7^-$	70i	10	Cl©$Sr_7Se_7^-$	64i	10
Cl©$Be_7Te_7^-$	86i	8	Cl©$Mg_7Te_7^-$	84i	10	Cl©$Ca_7Te_7^-$	72i	10	Cl©$Sr_7Te_7^-$	59i	10
Cl©$Be_7Po_7^-$	129i	10	Cl©$Mg_7Po_7^-$	84i	10	Cl©$Ca_7Po_7^-$	73i	10	Cl©$Sr_7Po_7^-$	59i	10
Br©$Be_7O_7^-$	35i	1	Br©$Mg_7O_7^-$	**6**	0	Br©$Ca_7O_7^-$	79i	10	Br©$Sr_7O_7^-$	58i	11
Br©$Be_7S_7^-$	49i	8	Br©$Mg_7S_7^-$	75i	10	Br©$Ca_7S_7^-$	58i	10	Br©$Sr_7S_7^-$	47i	10
Br©$Be_7Se_7^-$	81i	9	Br©$Mg_7Se_7^-$	39i	8	Br©$Ca_7Se_7^-$	56i	10	Br©$Sr_7Se_7^-$	45i	10
Br©$Be_7Te_7^-$	63i	10	Br©$Mg_7Te_7^-$	62i	10	Br©$Ca_7Te_7^-$	54i	10	Br©$Sr_7Te_7^-$	44i	10
Br©$Be_7Po_7^-$	57i	8	Br©$Mg_7Po_7^-$	53i	10	Br©$Ca_7Po_7^-$	50i	10	Br©$Sr_7Po_7^-$	47i	10
I©$Be_7O_7^-$	56i	1	I©$Mg_7O_7^-$	**25**	0	I©$Ca_7O_7^-$	77i	10	I©$Sr_7O_7^-$	52i	11
I©$Be_7S_7^-$	33i	8	I©$Mg_7S_7^-$	25i	4	I©$Ca_7S_7^-$	50i	10	I©$Sr_7S_7^-$	41i	10
I©$Be_7Se_7^-$	63i	8	I©$Mg_7Se_7^-$	44i	10	I©$Ca_7Se_7^-$	50i	10	I©$Sr_7Se_7^-$	42i	10
I©$Be_7Te_7^-$	51i	8	I©$Mg_7Te_7^-$	35i	8	I©$Ca_7Te_7^-$	45i	10	I©$Sr_7Te_7^-$	37i	10
I©$Be_7Po_7^-$	57i	10	I©$Mg_7Po_7^-$	32i	8	I©$Ca_7Po_7^-$	41i	10	I©$Sr_7Po_7^-$	37i	10
At©$Be_7O_7^-$	56i	1	At©$Mg_7O_7^-$	**16**	0	At©$Ca_7O_7^-$	71i	10	At©$Sr_7O_7^-$	57i	11
At©$Be_7S_7^-$	26i	4	At©$Mg_7S_7^-$	29i	8	At©$Ca_7S_7^-$	47i	10	At©$Sr_7S_7^-$	----	----
At©$Be_7Se_7^-$	21i	6	At©$Mg_7Se_7^-$	18i	4	At©$Ca_7Se_7^-$	41i	10	At©$Sr_7Se_7^-$	35i	10
At©$Be_7Te_7^-$	48i	10	At©$Mg_7Te_7^-$	33i	10	At©$Ca_7Te_7^-$	40i	10	At©$Sr_7Te_7^-$	31i	10
At©$Be_7Po_7^-$	33i	8	At©$Mg_7Po_7^-$	32i	10	At©$Ca_7Po_7^-$	38i	10	At©$Sr_7Po_7^-$	32i	10

**System**	**ν_{min}**	**NIMG**
$F©Ba_7O_7^-$	133i	15
$F©Ba_7S_7^-$	76i	13
$F©Ba_7Se_7^-$	-	-
$F©Ba_7Te_7^-$	74i	12
$F©Ba_7Po_7^-$	74i	10
**$Cl©Ba_7O_7^-$**	113i	17
$Cl©Ba_7S_7^-$	61i	10
$Cl©Ba_7Se_7^-$	-	-
$Cl©Ba_7Te_7^-$	59i	10
$Cl©Ba_7Po_7^-$	61i	10
$Br©Ba_7O_7^-$	111i	17
$Br©Ba_7S_7^-$	34i	10
$Br©Ba_7Se_7^-$	-	-
$Br©Ba_7Te_7^-$	43i	10
$Br©Ba_7Po_7^-$	43i	10
$I©Ba_7O_7^-$	111i	17
$I©Ba_7S_7^-$	40i	11
$I©Ba_7Se_7^-$	-	-
$I©Ba_7Te_7^-$	39i	14
$I©Ba_7Po_7^-$	42i	10
$At©Ba_7O_7^-$	110i	17
$At©Ba_7S_7^-$	30i	10
$At©Ba_7Se_7^-$	-	-
$At©Ba_7Te_7^-$	34i	14
$At©Ba_7Po_7^-$	27i	14

Table S3. Lowest vibrational frequencies (ν_{min}, cm^{-1}) of the D_{7h} Br©$Zn_7O_7^-$, I©$Zn_7O_7^-$, At©$Zn_7O_7^-$, and At©$Cd_7O_7^-$ systems, computed at different levels.

Level	Br©$Zn_7O_7^-$	I©$Zn_7O_7^-$	At©$Zn_7O_7^-$	At©$Cd_7O_7^-$
PBE0-D3/def2-TZVPP	4*i*	14	11	2
BP86-D3/def2-TZVPP	10*i*	12	6	2
B3LYP-D3/def2-TZVPP	13	7	10*i*	12
B3PW91-D3/def2-TZVPP	7	15	5	9
ωB97X-D/def2-TZVPP	10	15	4	6
TPSSh/def2-TZVPP	14*i*	7	13	9*i*
M06-2X/def2-TZVPP	10	10	14	11*i*

Table S4. HOMO-LUMO gap ($\Delta E_{H\text{-}L}$, eV), singlet-triplet energy gaps ($\Delta E_{S\text{-}T}$, kcal mol^{-1}), lowest vibrational frequencies (ν_{min}, cm^{-1}) and T_1 diagnostics for the global minimum Br©$Zn_7O_7^-$, I©$Zn_7O_7^-$, At©$Zn_7O_7^-$, and At©$Cd_7O_7^-$. Energy gaps and vibrational frequencies were computed at the ωB97X-D/def2-TZVPP level, whereas T_1 diagnostics were obtained at the CCSD/CBS level of theory.

System	$\Delta E_{H\text{-}L}$	$\Delta E_{S\text{-}T}$	ν_{min}	T_1
Br©$Zn_7O_7^-$	8.8	41.3	10	0.011
I©$Zn_7O_7^-$	8.5	48.5	16	0.011
At©$Zn_7O_7^-$	8.2	58.2	4	0.011
At©$Cd_7O_7^-$	7.5	42.0	6	0.015

Table S5. EDA results for Br©$Zn_7O_7^-$, I©$Zn_7O_7^-$, At©$Zn_7O_7^-$, and At©$Cd_7O_7^-$ using differently charged fragments at the PBE0/TZ2P-ZORA level. $\Delta E_{att} = \Delta E_{elstat} + \Delta E_{orb}$. Energies are given in kcal·mol^{-1}. (S: singlet; D: doublet; T: triplet).

	**Br^-(S) + Zn_7O_7(S)**	**Br(D) + $Zn_7O_7^-$(D)**	**Br^+(S) + $Zn_7O_7^{2-}$ (S)**	**Br^+(T) + $Zn_7O_7^{2-}$ (T)**
ΔE_{int}	−67.6	−220.8	−494.4	−459.4
ΔE_{Pauli}	26.1	46.2	86.3	50.2
ΔE_{att}	−93.7	−266.9	−580.7	−509.6
ΔE_{elstat}[a]	−70.7 (75.5%)	−18.7 (7.0%)	−184.9 (31.8%)	−158.7 (31.1%)
ΔE_{orb}[a]	**−23.0 (24.5%)**	−248.3 (93.0%)	−395.8 (68.2%)	−350.9 (68.9%)

	**I^-(S) + Zn_7O_7(S)**	**I(D) + $Zn_7O_7^-$(D)**	**I^+(S) + $Zn_7O_7^{2-}$ (S)**	**I^+(T) + $Zn_7O_7^{2-}$ (T)**
ΔE_{int}	−62.1	−208.9	−445.9	−417.9
ΔE_{Pauli}	48.6	69.5	121.2	72.0
ΔE_{att}	−110.8	−278.4	−567.1	−489.9
ΔE_{elstat}[a]	−86.4 (78.0%)	−30.3 (10.9%)	−205.6 (36.2%)	−171.8 (35.1%)
ΔE_{orb}[a]	**−24.4 (22.0%)**	−248.1 (89.1%)	−361.6 (63.8%)	−318.2 (64.9%)

	**At^-(S) + Zn_7O_7(S)**	**At(D) + $Zn_7O_7^-$(D)**	**At^+(S) + $Zn_7O_7^{2-}$ (S)**	**At^+(T) + $Zn_7O_7^{2-}$ (T)**
ΔE_{int}	−60.9	−201.6	−422.2	−396.3
ΔE_{Pauli}	61.5	80.5	137.2	82.2
ΔE_{att}	−122.6	−282.1	−559.4	−478.6
ΔE_{elstat}[a]	−96.8 (79.0%)	−38.1 (13.5%)	−221.1 (39.5%)	−181.2 (37.9%)
ΔE_{orb}[a]	**−25.8 (21.0%)**	−244.0 (86.5%)	−338.3 (60.5%)	−297.4 (62.1%)

	**At^-(S) + Cd_7O_7(S)**	**At(D) + $Cd_7O_7^-$(D)**	**At^+(S) + $Cd_7O_7^{2-}$ (S)**	**At^+(T) + $Cd_7O_7^{2-}$ (T)**
ΔE_{int}	−58.3	−108.2	−407.9	−379.3
ΔE_{Pauli}	35.5	44.2	83.2	48.4
ΔE_{att}	−93.8	−152.4	−491.2	−427.7
ΔE_{elstat}[a]	−73.3 (78.1%)	−35.2 (22.2%)	−175.9 (35.8%)	−150.2 (35.1%)
ΔE_{orb}[a]	**−20.5 (21.9%)**	−117.2 (76.8%)	−315.3 (64.2%)	−277.5 (64.9%)
$\Delta E_{orb(1)}$[a]	−6.7 (32.7%)			
$\Delta E_{orb(2)}$[b]	−6.7 (32.7%)			
$\Delta E_{orb(3)}$[b]	−1.1 (5.4%)			
$\Delta E_{orb(rest)}$[b]	−6.0 (29.2%)			

[a]The percentage contribution relative to the total attractive interaction is given in parentheses.
[b]The percentage contribution relative to the total orbital interaction is given in parentheses.

Table S6. IQA energy components for the D_{7h} Br©$Zn_7O_7^-$, I©$Zn_7O_7^-$, At©$Zn_7O_7^-$ and At©$Cd_7O_7^-$ systems at the PBE0/def2-TZVPP//ωB97X-D/def2-TZVPP level. ΔE_{IQA} is the total integration error in the IQA energies; V_{IQA}^{int}, is the interatomic IQA energy, and V_{C}^{int}, and V_{XC}^{int} are its Coulombic and exchange-correlation components, respectively. Energies are given in kcal mol^{-1}.

	**Br©$Zn_7O_7^-$**	**I©$Zn_7O_7^-$**	**At©$Zn_7O_7^-$**	**At©$Cd_7O_7^-$**
ΔE_{IQA}	0.0	0.0	0.0	0.0
V_{IQA}^{int}(X–M)	–95.5	–87.5	–82.1	–73.5
V_{C}^{int}(X–M)	–87.7	–77.3	–70.9	–64.3
V_{XC}^{int}(X–M)	–7.9	–10.1	–11.2	–9.2
V_{IQA}^{int}(M–M)	163.2	160.0	158.0	119.4
V_{C}^{int}(M–M)	166.2	162.9	160.9	122.9
V_{XC}^{int}(M–M)	–3.0	–2.9	–2.9	–3.5
V_{IQA}^{int}(M–O)	–391.9	–389.3	–388.0	–326.7
V_{C}^{int}(M–O)	–268.6	–267.3	–266.4	–206.4
V_{XC}^{int}(M–O)	–123.2	–122.0	–121.6	–120.3

Table S7. Theoretical first vertical detachment energies (VDEs) of the D_{7h} Br©$Zn_7O_7^-$, I©$Zn_7O_7^-$, At©$Zn_7O_7^-$ and At©$Cd_7O_7^-$ systems computed at the OVGF/def2-TZVPP//ωB97X-D/def2-TZVPP, DLPNO-CCSD(T)/CBS//ωB97X-D/def2-TZVPP, and TD-ωB97X-D/def2-TZVPP//ωB97X-D/def2-TZVPP levels of theory. All energies are given in eV. Values in parentheses are OVGF pole strengths.

**System**	**OVGF**	**CCSD(T)**	**TD-ωB97X-D**
Br©$Zn_7O_7^-$	6.39 (0.90)	6.55	6.32
I©$Zn_7O_7^-$	5.78 (0.90)	5.83	5.92
At©$Zn_7O_7^-$	5.51 (0.91)	8.04	5.67
At©$Cd_7O_7^-$	5.24 (0.91)	5.38	5.50

Table S8. Cartesian coordinates of the Br©$Zn_7O_7^-$, I©$Zn_7O_7^-$, At©$Zn_7O_7^-$, At©$Cd_7O_7^-$, Zn_7O_7, Cd_7O_7, F©$Zn_5O_5^-$, and Cl©$Zn_6O_6^-$ systems optimized at the ωB97X-D/def2-TZVPP level.

**Br©$Zn_7O_7^-$**				**I©$Zn_7O_7^-$**			
O	1.760293000	3.655286000	0.000000000	O	1.765781000	3.666682000	0.000000000
O	3.955343000	0.902781000	0.000000000	O	3.967674000	0.905596000	0.000000000
O	3.171938000	-2.529537000	0.000000000	O	3.181827000	-2.537422000	0.000000000
O	0.000000000	-4.057062000	0.000000000	O	0.000000000	-4.069710000	0.000000000
O	-3.171938000	-2.529537000	0.000000000	O	-3.181827000	-2.537422000	0.000000000
O	-3.955343000	0.902781000	0.000000000	O	-3.967674000	0.905596000	0.000000000
O	-1.760293000	3.655286000	0.000000000	O	-1.765781000	3.666682000	0.000000000
Zn	0.000000000	3.497901000	0.000000000	Zn	0.000000000	3.525442000	0.000000000
Zn	-2.734769000	2.180905000	0.000000000	Zn	-2.756301000	2.198077000	0.000000000
Zn	-3.410201000	-0.778356000	0.000000000	Zn	-3.437052000	-0.784485000	0.000000000
Zn	-1.517682000	-3.151500000	0.000000000	Zn	-1.529632000	-3.176313000	0.000000000
Zn	1.517682000	-3.151500000	0.000000000	Zn	1.529632000	-3.176313000	0.000000000
Zn	3.410201000	-0.778356000	0.000000000	Zn	3.437052000	-0.784485000	0.000000000
Zn	2.734769000	2.180905000	0.000000000	Zn	2.756301000	2.198077000	0.000000000
Br	0.000000000	0.000000000	0.000000000	I	0.000000000	0.000000000	0.000000000

**At©$Zn_7O_7^-$**				**At©$Cd_7O_7^-$**			
O	1.767615000	3.670490000	0.000000000	O	1.952032000	4.053436000	0.000000000
O	3.971794000	0.906536000	0.000000000	O	4.386176000	1.001116000	0.000000000
O	3.185131000	-2.540058000	0.000000000	O	3.517440000	-2.805065000	0.000000000
O	0.000000000	-4.073936000	0.000000000	O	0.000000000	-4.498975000	0.000000000
O	-3.185131000	-2.540058000	0.000000000	O	-3.517440000	-2.805065000	0.000000000
O	-3.971794000	0.906536000	0.000000000	O	-4.386176000	1.001116000	0.000000000
O	-1.767615000	3.670490000	0.000000000	O	-1.952032000	4.053436000	0.000000000
Zn	0.000000000	3.533355000	0.000000000	Cd	0.000000000	3.868779000	0.000000000
Zn	-2.762488000	2.203011000	0.000000000	Cd	-3.024733000	2.412144000	0.000000000
Zn	-3.444767000	-0.786246000	0.000000000	Cd	-3.771781000	-0.860884000	0.000000000
Zn	-1.533065000	-3.183443000	0.000000000	Cd	-1.678600000	-3.485650000	0.000000000
Zn	1.533065000	-3.183443000	0.000000000	Cd	1.678600000	-3.485650000	0.000000000
Zn	3.444767000	-0.786246000	0.000000000	Cd	3.771781000	-0.860884000	0.000000000
Zn	2.762488000	2.203011000	0.000000000	Cd	3.024733000	2.412144000	0.000000000
At	0.000000000	0.000000000	0.000000000	At	0.000000000	0.000000000	0.000000000

**Zn_7O_7**				**Cd_7O_7**			
O	1.757858000	3.650229000	0.000000000	O	1.949990000	4.049195000	0.000000000
O	3.949871000	0.901532000	0.000000000	O	4.381587000	1.000069000	0.000000000
O	3.167550000	-2.526037000	0.000000000	O	3.513760000	-2.802130000	0.000000000
O	0.000000000	-4.051449000	0.000000000	O	0.000000000	-4.494268000	0.000000000
O	-3.167550000	-2.526037000	0.000000000	O	-3.513760000	-2.802130000	0.000000000
O	-3.949871000	0.901532000	0.000000000	O	-4.381587000	1.000069000	0.000000000
O	-1.757858000	3.650229000	0.000000000	O	-1.949990000	4.049195000	0.000000000
Zn	0.000000000	3.634284000	0.000000000	Cd	0.000000000	3.989347000	0.000000000
Zn	-2.841398000	2.265939000	0.000000000	Cd	-3.118997000	2.487317000	0.000000000
Zn	-3.543165000	-0.808704000	0.000000000	Cd	-3.889326000	-0.887713000	0.000000000
Zn	-1.576857000	-3.274377000	0.000000000	Cd	-1.730913000	-3.594277000	0.000000000

Zn	1.576857000	-3.274377000	0.000000000	Cd	1.730913000	-3.594277000	0.000000000
Zn	3.543165000	-0.808704000	0.000000000	Cd	3.889326000	-0.887713000	0.000000000
Zn	2.841398000	2.265939000	0.000000000	Cd	3.118997000	2.487317000	0.000000000
**F©$Zn_5O_5^-$**				**Cl©$Zn_6O_6^-$**			
O	1.789378000	2.462868000	0.000000000	Cl	0.000000000	0.000000000	0.000000000
F	0.000000000	0.000000000	0.000000000	Zn	0.000000000	2.969224000	0.000000000
Zn	0.000000000	2.400398000	0.000000000	Zn	2.572320000	1.485130000	0.000000000
Zn	-1.410919000	-1.941963000	0.000000000	Zn	-2.572320000	1.485130000	0.000000000
Zn	2.282914000	0.741764000	0.000000000	Zn	-2.571424000	-1.484612000	0.000000000
O	0.000000000	-3.044272000	0.000000000	Zn	0.000000000	-2.970259000	0.000000000
O	-1.789378000	2.462868000	0.000000000	Zn	2.571424000	-1.484612000	0.000000000
O	-2.895275000	-0.940732000	0.000000000	O	3.554526000	-0.000387000	0.000000000
O	2.895275000	-0.940732000	0.000000000	O	1.776928000	3.078503000	0.000000000
Zn	1.410919000	-1.941963000	0.000000000	O	-1.776928000	3.078503000	0.000000000
Zn	-2.282914000	0.741764000	0.000000000	O	-3.554526000	-0.000387000	0.000000000
				O	-1.777598000	-3.078116000	0.000000000
				O	1.777598000	-3.078116000	0.000000000
